# Countries’ Biomedical Publications and Attraction Scores. A PubMed-based assessment

**DOI:** 10.12688/f1000research.5775.2

**Published:** 2015-08-11

**Authors:** Qinyi Xu, Andrea Boggio, Andrea Ballabeni

**Affiliations:** 1 Bentley University, Waltham, MA, 02452, USA; 2 Department of History and Social Science, Bryant University, Smithfield, RI, 02917, USA; 3 Department of Health Policy and Management, Harvard School of Public Health, Boston, MA, 02115, USA

**Keywords:** bibliometrics, Attraction Score, publications, PubMed

## Abstract

Studying publication volumes at the country level is key to understanding and improving a country’s research system. PubMed is a public search engine of publications in all life sciences areas. Here, we show how this search engine can be used to assess the outputs of life science-related research by country. We have measured the numbers of publications during different time periods based on the country of affiliation of the first authors. Moreover, we have designed scores, which we have named Attraction Scores, to appraise the relative focus either toward particular types of studies, such as clinical trials or reviews, or toward specific research areas, such as public health and pharmacogenomics, or toward specific topics, for instance embryonic stem cells; we have also investigated a possible use of these Attraction Scores in connection with regulatory policies. We have weighed the statistics against general indicators such as country populations and gross domestic products (GDP). During the 5-year period 2008-2012, the United States was the country with the highest number of publications and Denmark the one with the highest number of publications *per capita*. Among the 40 countries with the highest GDPs, Israel had the highest publications-to-GDP ratio. Among the 20 countries with the most publications, Japan had the highest Attraction Score for induced pluripotent stem (iPS) cells and Italy the highest proportion of review publications. More than 50% of publications in English were from countries in which English is not the primary language. We show an assorted and extensive collection of rankings and charts that will inform scholars and policymakers in studying and improving the research systems both at the national and international level.

## Introduction

Publication output at the country level has been assessed by means of different tools and methodologies. Both academic groups and private companies, by using different and complementary approaches, have offered valuable information to scholars and policymakers ^1– 6^. Some of these efforts have focused specifically on the life science sector by taking advantage of PubMed ( http://www.ncbi.nlm.nih.gov/pubmed/) ^6– 9^, a free and public search engine that provides access to over 24 million citations in all fields of life sciences, mostly located in the MEDLINE (Medical Literature Analysis and retrieval System Online) bibliographic database. PubMed became accessible to the public at no charge in June 1997 and has been maintained by the United States National Library of Medicine (NLM) at the National Institutes of Health (NIH). Over the years, it has become a very popular database among scientists and nonscientists interested in retrieving research publications in various biological and biomedical fields. One of the most valuable features is users’ ability to restrict searches through different filters and settings. Users can target their searches by restricting the query to publications that meet various requirements—including time of publication, type of publication, type of research, language, authors’ affiliation, journal, and other criteria—and by searching for specific keywords in the full text of the article or in sub-parts, such as title and abstract. The system uses the Boolean operators AND, OR and NOT to facilitate filtering and refining of the searches.

With the goal of assisting scholars and policymakers in studying and improving research systems at the national and international level, we present a methodology that deploys PubMed to assess “bio” publication output and the sharing of publications for certain types of research and topics of interest. We also present data that can be retrieved using this methodology. Though PubMed search engine and MEDLINE database had been previously used to assess the quantity of research publications of countries ^7– 11^, our study constitutes the most recent, assorted and refined assessment of publication output in the life sciences. Thanks to the ability to attribute a paper to various countries, we present data of publication output by country and supranational regions. Further, we have used various stringency criteria in order to check the method’s robustness. Thus, we have analyzed publication output in different time ranges and calculated publication output in relation to various country-specific statistics (populations, GDPs, research and development (R&D) expenditures and presence/absence of English as a primary language). Finally, we have created a new score (Attraction Score) that measures the relative weight of publications for certain kinds of research or certain topics of interest. By analyzing the relationship between the Attraction Scores for human embryonic stem cells and the regulatory policies, we propose an example of how these Attraction Scores could be used to assess the research impact of regulatory policies. We believe that the methodology and the graphic representations of data will provide valuable and easy-to-grasp information that can assist professionals and the general public in understanding and improving biomedical research governance.

## Methods

### Publications by country and globally

The number of publications by country was determined by inserting the name/s of the countries in the “affiliation” field in the “advanced” section of the PubMed search engine. In the event of publications with authors based in different countries, we chose to attribute the paper only to the country of the leading author rather than collaborators. This was facilitated by the fact that PubMed only reports affiliation information of the first author of articles published before 2014 ( http://www.ncbi.nlm.nih.gov/books/NBK3827/#pubmedhelp.Affiliation_AD) (see also Discussion section). Since this study focuses mainly on publications from 1993 to 2012, we have attributed a paper to a certain country based on the first author’s affiliation. We do not believe that this is a problem: in the vast majority of cases, first author and corresponding author are either the same person or they work in the same research institution/geographical area and therefore have the same country affiliation. To address the problem of countries recorded under multiple names, we have used the different designations separated by the Boolean operator OR. For example, publications of the United Kingdom were searched by inserting “united kingdom OR uk” in the “affiliation” field. Publications of the United States were searched by inserting “us OR usa OR united states” (we observed only small differences when inserting “usa OR united states”, see Discussion section). To identify the year of publication, we used the “Custom range” function (that is equivalent to using the “year:year[dp]” syntax in the search field). Most of the searches were made for the 5-years period 2008–2012, but we also investigated the 5-years periods 1993–1997, 1998–2002, and 2003–2007 to determine changes in publications volumes over time. Searches were also performed for individual years by using the “Custom range” function or by using the CSV downloads of the automatically retrieved yearly counts. We constrained publication output primarily on papers reporting original research. Therefore, unless otherwise indicated, we excluded reviews from our queries. We did so by using the operator NOT before the word “review” typed in the “Publication Type” field.

In generating publication data by continents, we added the number of publications of all countries in a certain continent. America was divided into North-Central America and South America. The publications of Russia and Turkey were allocated half to Asia and half to Europe. With regard to the European Union, we added the number of publications of the 28 countries that have joined the EU to present day. It should be noted, however, that some of the countries joined the European Union between 1993 and 2012. (One country, Croatia, joined the EU in 2013, and thus, it was not part of the EU at any time during the time periods under investigation). The total numbers of publications for the entire world were calculated by leaving the affiliation fields blank.

In addition to the standard criteria discussed so far (criteria A), we also conducted searches using various levels of stringency to criteria A. We thus added the “Journal Article” filter (in “Article types”) and “English” (in “Languages”) to criteria A to generate criteria B; the “Journal Article” filter (in “Article types”) to generate criteria C; the “English” filter (in “Languages”) to generate criteria D. The percentages of publications written in English were estimated by dividing the number of publications obtained using criteria D by the number of publications obtained using criteria A and then by multiplying the result by 100. The countries considered to have English as primary languages are shown in the data file. We observed only small differences between the counts obtained by using these four criteria (See Results section). We also decided to identify papers that reported the results of clinical trials. To this end, we created criteria E by adding the “Clinical Trial” filter to criteria A. This way, we were able to retrieve only publications based on clinical trial studies.

### Attraction Scores

We chose to retrieve publications in certain specific areas or that discuss specific topics. To this end, we inserted in quotes the chosen research areas or topics of interest (for example, “public health” or “personalized medicine”) in the “Title/Abstract” field in the “Advanced” section of PubMed. This step allowed as to generate Attraction Scores for areas or topics (named “ *Area* or *Topic* Attraction Scores”), which were calculated by dividing the number of publications in certain research areas or discussing certain topics, in a certain country, and within a certain period, by the total number of publications in the same country/period and by multiplying it by 10,000 to obtain easy-to-read numbers. The Area/Topic Attraction Scores were calculated by using criteria B because, as the searched areas or topics were English words, we wanted to exclude the (few) publications written in languages other than English. Nonetheless, we run the same query also using criteria A, and we obtained very similar results. Since the results are irrelevant, we decided not to show data of queries with criteria A in this paper. In the case of the “human embryonic stem cells” topic, we also used a “hESC/ESC Score”, defined as the ratio between number of publications obtained by using the search term “human embryonic stem cells” in the “Title/Abstract” field and the number of publications obtained by using the search term “embryonic stem cells” in the “Title/Abstract” field.

Similarly, we also generated “Clinical Trial Attraction Scores” by dividing the numbers of publications based on clinical trials studies retrieved using Criteria E by the total number of publications, retrieved using criteria A, in the same country and in the same period, and by multiplying the result by 10,000.

Finally, we also generated Review Attraction Scores by comparing the numbers of publications obtained by criteria A with the numbers of publications obtained by criteria A+Reviews (i.e. without the exclusion of reviews) and by multiplying the result by 100 (in this case the score is the percentage).

### Publications relative to population, GDP, and R&D expenditure

We studied publication output during the 5-year period 2008–2012 relative to population, gross domestic products (GDP), and R&D expenditure. Population data ( http://data.worldbank.org/indicator/SP.POP.TOTL) and GDP data ( http://data.worldbank.org/indicator/NY.GDP.MKTP.CD) were retrieved from World Bank databases. We chose 2011 as a reference year. R&D expenditure data were retrieved from Battelle (a private nonprofit science and technology development company) 2014 Global R&D Funding Forecast ( http://www.battelle.org/docs/tpp/2014_global_rd_funding_forecast.pdf) and expressed as GERD (Gross Expenditure on Research and Development) of year 2012 with Purchasing Power Parity in US$ billion. Data on population by continent were obtained from estimates published by the US Census Bureau ( http://www.census.gov/compendia/statab/2012/tables/12s1330.pdf) for the year 2010. The numbers of total citable documents by country were retrieved from the SCImago Journal & Country Rank ( http://www.scimagojr.com/index.php).

### Other methodological notes

We repeated several searches one year apart to evaluate the consistency of the results across time. Several searches were therefore run a first time in July/August of 2013 and then repeated in September/October of 2014. We obtained very similar results, including for the year 2012 (See Results section). This shows that PubMed is rapidly updated and stable across time.

The classification of policies that regulate the use of human embryonic stem cells is based on the one previously developed by the Hinxton Group ( http://www.hinxtongroup.org), a consortium fostering international cooperation in stem cell research. Other sources for country-specific policies are referenced in the Results section.

## Results

Biomedical publication and Attraction Score data based on PubMed searches The database contains data about the volumes of biomedical publications by country. Review publications are generally excluded, unless otherwise specified. The data refer either to the general publications, the clinical trial-based publications or the publications on certain research areas or topics. Attraction Scores indicate the proportions of publications on certain research areas or topics relative to the total volumes of publications. The numbers of publications are also compared to general non-bibliometric indicators such as the country population, the GDP and the R&D expenditure. Database also contains estimates of the publications in English. Statistics about supra-national entities like continents or the European Union is also included. The volumes of publications were determined by using the PubMed search engine. The data refer to time periods 1993-1997, 1998-2002, 2003-2007, 2008-2012 or the single year 2012. Several searches were made a first time in July-August 2013 and then repeated in September/October 2014 to check for the consistency of data over time. The data are ranked according to different criteria. Columns/row headings describe the contents of the columns/rows. A detailed description of the methodology and of the results is contained in the associated article.
Only the first 15 sheets are pre-viewable, please download the file to see the complete database. Click here for additional data file. Copyright: © 2015 Xu Q et al. 2015 Data associated with the article are available under the terms of the Creative Commons Zero "No rights reserved" data waiver (CC0 1.0 Public domain dedication).

### The vast majority of publications contain a country name in the affiliation

**Figure S1.**
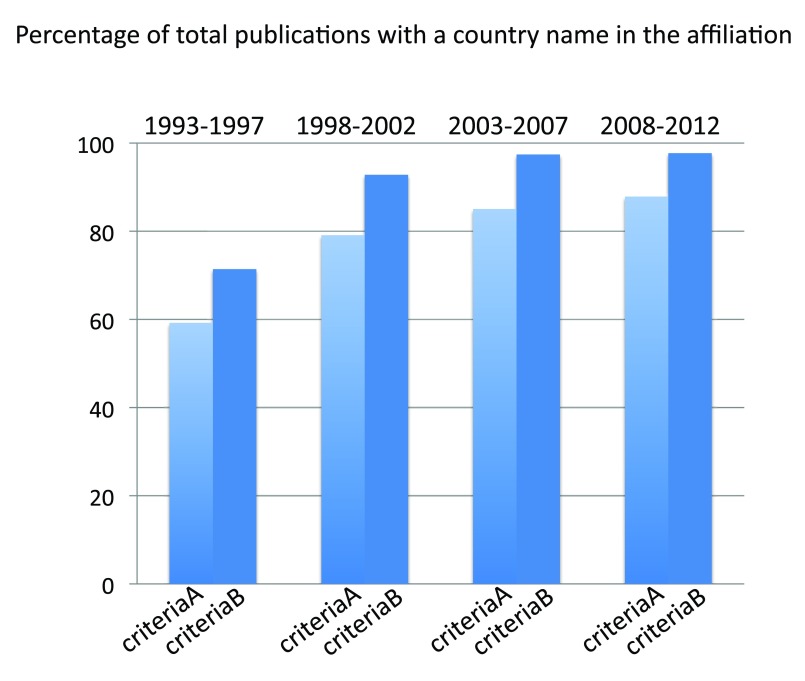
Percentages of publications (reviews excluded) with the country name in the affiliation field. Criteria A and B were used to calculate the proportions in the 5-year periods 1993–1997, 1998–2002, 2003–2007, and 2008–2012. The total world publications (reviews excluded) were obtained by searches without any specification in the affiliation field. The total world publications (reviews excluded) with the country name in the affiliation field were obtained by summing the numbers of publications (reviews excluded) of all countries of the world.

We determined the number of publications of countries for the 5-year periods 1993–1997, 1998–2002, 2003–2007, and 2008–2012. We divided the sums of publications for all countries by the total numbers of world publications (obtained without searching any country name in the “Affiliation” field, see Methods section), in the same time periods. We used both criteria A and criteria B for this analysis. We observed that the percentage of publications containing a country name in the affiliation increases with time. In the time period 2008–2012, the proportion of papers with a country name in the affiliation is 87.8% and 97.7% by using criteria A and criteria B, respectively ( Figure S1) (sheet 1, including also the number of publications for all the countries for the time periods 2003–2007 and 2008–2012) (sheets of the database show results either from 2013 or 2014 searches. When not specified, the searches were made in 2013. When not specified, searches were for the time period 2008–2012). These data indicate that the methodology can plausibly effectively estimate the volumes of life science and biomedical publications of countries.

### The volume of publications has increased at a constant rate over 20 years

**Figure 1.**
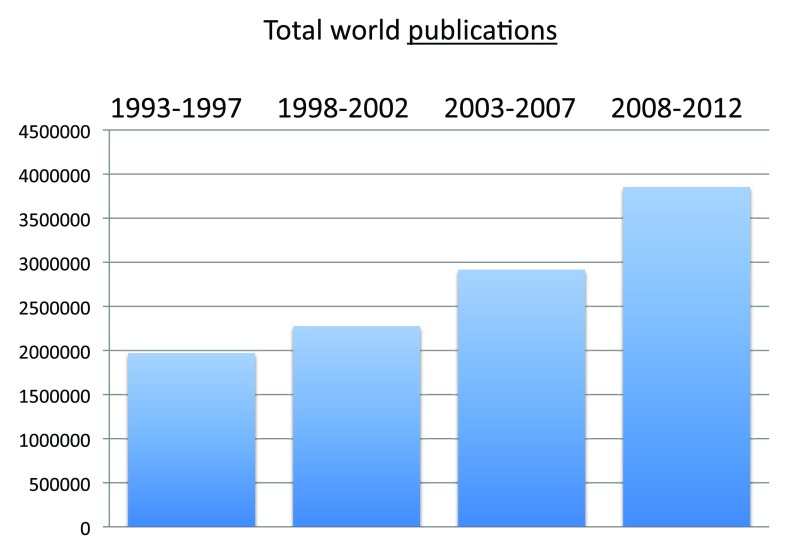
Total world publications (reviews excluded) in the 5-year periods 1993–1997, 1998–2002, 2003–2007, and 2008–2012. Criteria A.

We determined the number of total world publications (obtained without searching any country name in the “Affiliation” field, see Methods section) for the time periods 1993–1997, 1998–2002, 2003–2007, and 2008–2012. Publications increased with a nearly constant rate during the four time periods, with the volume of the time period 2008–2012 corresponding to over 3.8 million publications (reviews excluded according to our above described searching criteria), roughly the double of the volume of the time period 1993–1997 ( Figure 1) (sheet 2).

### The proportion of clinical trial studies has remained almost stable in 20 years

**Figure 2.**
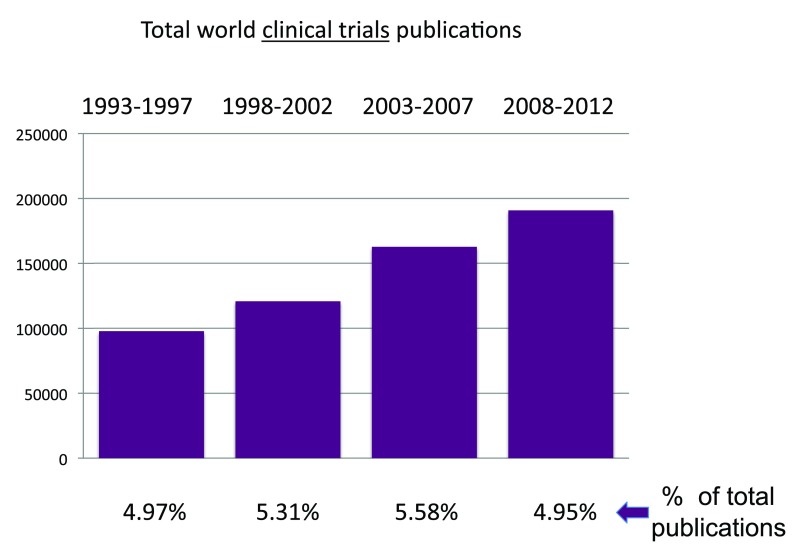
Total world Clinical Trial publications (reviews excluded) in the 5-year periods 1993–1997, 1998–2002, 2003–2007, and 2008–2012. The proportions of publications that are Clinical Trial publications are shown. Criteria E.

We determined the number of world publications based on clinical trial studies (obtained without searching any country name in the “Affiliation” field, see Methods section), for the time periods 1993–1997, 1998–2002, 2003–2007, and 2008–2012. Also, this type of publication increased at a nearly constant rate throughout this time span. Interestingly, the share of publications based on clinical trial studies has remained nearly constant (close to 5% of total publications) during this time span ( Figure 2) (sheet 2).

### The proportion of publications in English has progressively increased during the last 20 years

**Figure S2.**
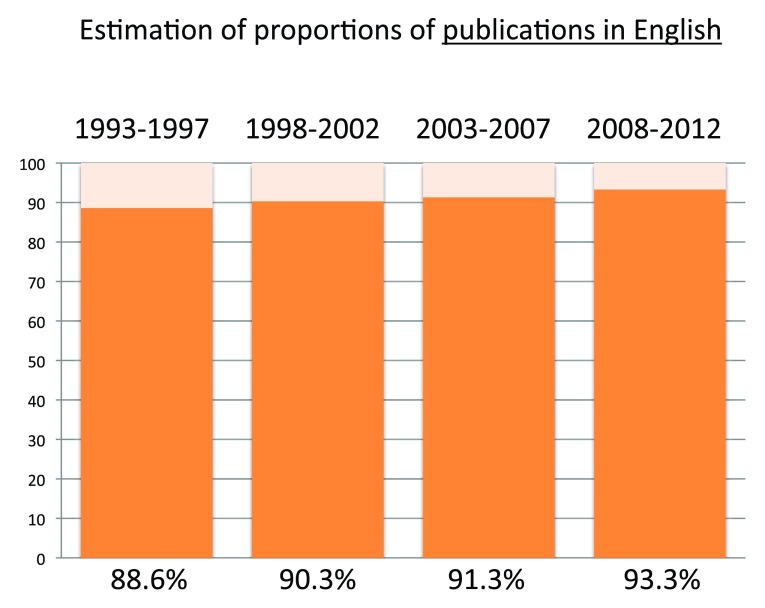
Estimation of the percentages of publications (reviews excluded) written in English in the 5-year periods 1993–1997, 1998–2002, 2003–2007, and 2008–2012. The percentages were calculated by determining the proportions between the numbers of publications obtained with criteria D and the numbers of publications obtained with criteria A.

We estimated the share of world publications written in English by calculating the ratio between the numbers of publications determined by criteria D and the numbers of publications determined by criteria A for the time periods 1993–1997, 1998–2002, 2003–2007, and 2008–2012. The number of publications in English slightly increased during the four time periods, being 88.6% in the time period 1993–1997 and 93.3% in the time period 2008–2012 ( Figure S2) (sheet 2).

### The United States is the country with more publications

**Figure 3.**
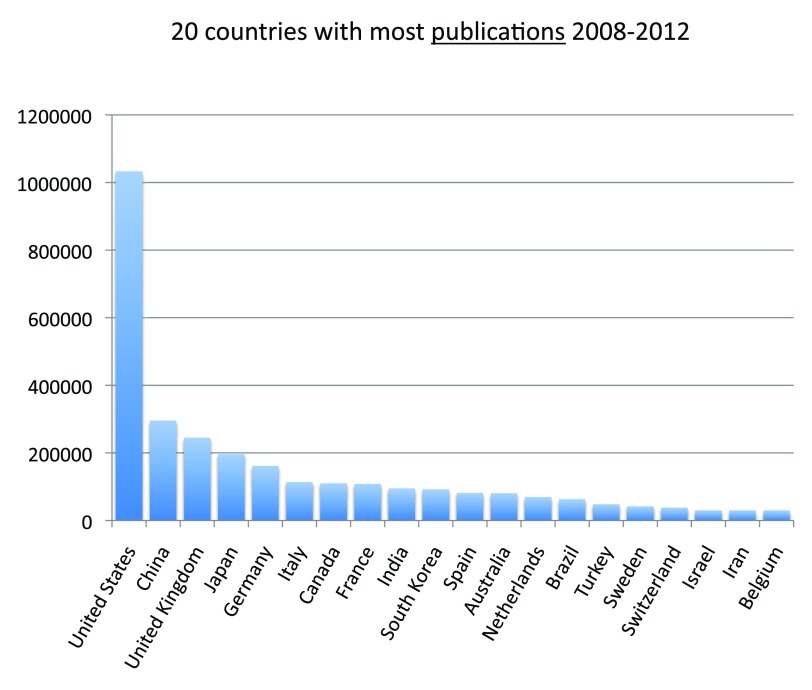
Numbers of publications (reviews excluded) for the 20 countries with the most publications (reviews excluded) in the 5-year period 2008–2012. Criteria A.

**Figure 4.**
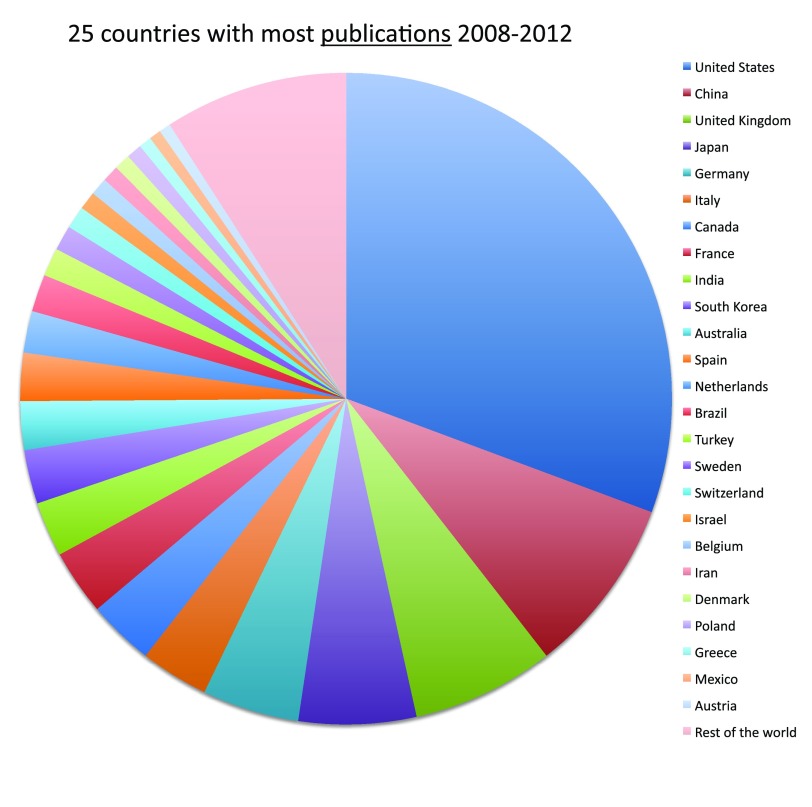
Numbers of publications (reviews excluded) for the 25 countries with the most publications (reviews excluded) in the 5-year period 2008–2012. The part of publications other than the top 25 countries is shown as “rest of the world” (in pale red). In this case, differently from Figure 3, the data were obtained from 2013 searches. Criteria A.

**Figure S3.**
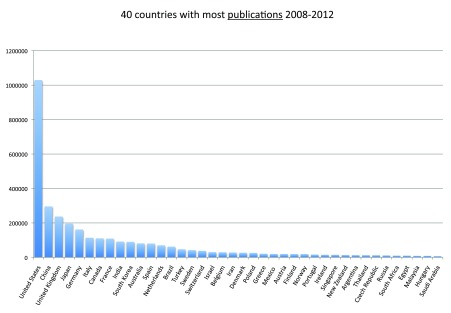
Numbers of publications (reviews excluded) of the 40 countries with the most publications (reviews excluded) in the 5-year period 2008–2012. In this case, differently from Figure 3, the data were obtained from 2013 searches. Criteria A.

We ranked all the countries according to their numbers of publications. In Figure 3 and Figure S3 (sheets 3 and 4), we show the charts with the 20 and 40 countries, respectively, with the most publications in the time period 2008–2012. With over one million publications, the United States represents by far the country with more publications than any other country, representing almost one-third of all world publications during the time period 2008–2012. The second-ranked country is China with a share of publication that is 28.5% of those attributed to the United States. In Figure 4 (sheet 4), we show a pie chart with the 25 countries with the most publications, including a “slice” representing the rest of the world (representing 10.0% of the total publications). We also tested the four different criteria (see Methods section) to determine this ranking. We noticed only minor differences in the numbers. The relative standard deviation was, on average, 1.9% for the 20 countries with the most publications (sheet 3). The only substantial differences between the four different criteria were for China (with a relative standard deviation equal to 13.7%) and France (with a relative standard deviation equal to 7.1%). These differences can be attributed primarily to the activation of the English language filter (See results below).

### Search results are consistent over time

In order to control for the consistency of these data over time, we ran several searches at two separate times at least one year apart (July/August 2013 and September/October 2014). In sheet 3 we show the differences in the total world publications (i.e. no affiliation specification) for the four different criteria. The differences were very small: below 1.5% for all four criteria. We also compared 2013 vs 2014 searches for the publications of countries with the most publications. Even in this case the results were very similar, and the average difference for the 20 countries with the most publications in the time period 2008–2012 was only 0.6%, with a maximum difference for Iran (6.1%) (sheet 4). These data confirm that PubMed is a reliable search engine that accurately retrieves information from databases that are promptly updated.

### The vast majority of papers of the 20 countries with the most publications are written in English

**Figure S4.**
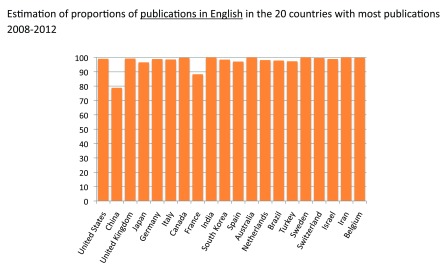
Estimation of the percentages of publications (reviews excluded) written in English of the 20 countries with the most publications (reviews excluded) in the 5-year period 2008–2012. The percentages were calculated by determining the proportions between the numbers of publications obtained with criteria D and the numbers of publications obtained with criteria A.

Using the same method described above, we estimated the percentage of publications in English for the 20 countries with the most publications in the time period 2008–2012. With the exceptions of France (88.3%) and China (78.8%), more than 95% of publications of all other countries were written in English ( Figure S4) (sheet 3). The PubMed search engine seems to be fairly accurate in the classification of languages of articles; indeed, we saw that the proportions of publications in English of Anglophone countries like the United States, the United Kingdom and Australia were over 99%.

### About 10% of publications in the 20 countries with the most publications are reviews

**Figure S5.**
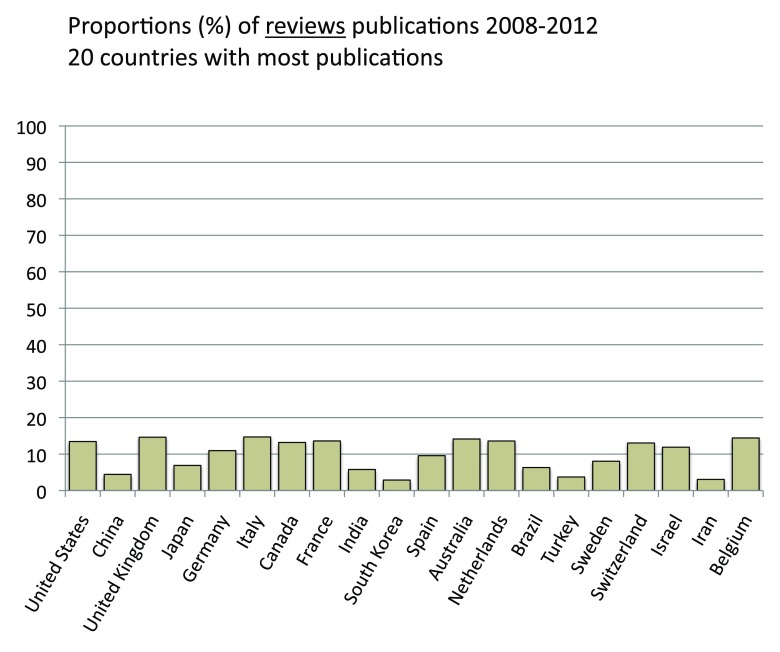
Estimation of the percentages of review publications of the 20 countries with the most publications (reviews excluded) in the 5-year period 2008–2012. The percentages were calculated by determining the proportion between the numbers of publications obtained with Criteria A and the numbers of publications obtained with Criteria A but without the exclusion of the reviews.

We also determined the proportion of review publications for the 20 countries with the most publications in the time period 2008–2012. They were calculated by comparing searches with or without the exclusion of reviews in the “Publication Types” field (See Methods section). On average, review publications constituted 9.9% of the publications, ranging from a maximum of 14.7% (Italy) to a minimum of 2.9% for South Korea ( Figure S5) (sheet 4).

### Denmark has the highest publications *per capita*

**Figure 5.**
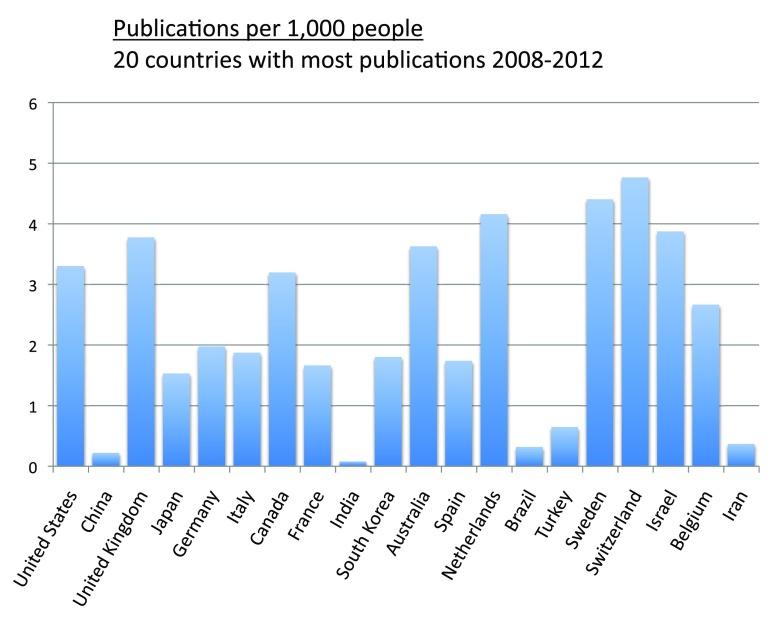
Publications (reviews excluded) *per capita* in the 20 countries with the most publications (reviews excluded) in the 5-year period 2008–2012. The numbers represent publications per 1,000 people and were obtained by dividing the number of publications (reviews excluded) by the country populations and multiplying by 1,000. In this case, differently from Figure 3, the data were obtained from 2013 searches. Criteria A.

**Figure 6.**
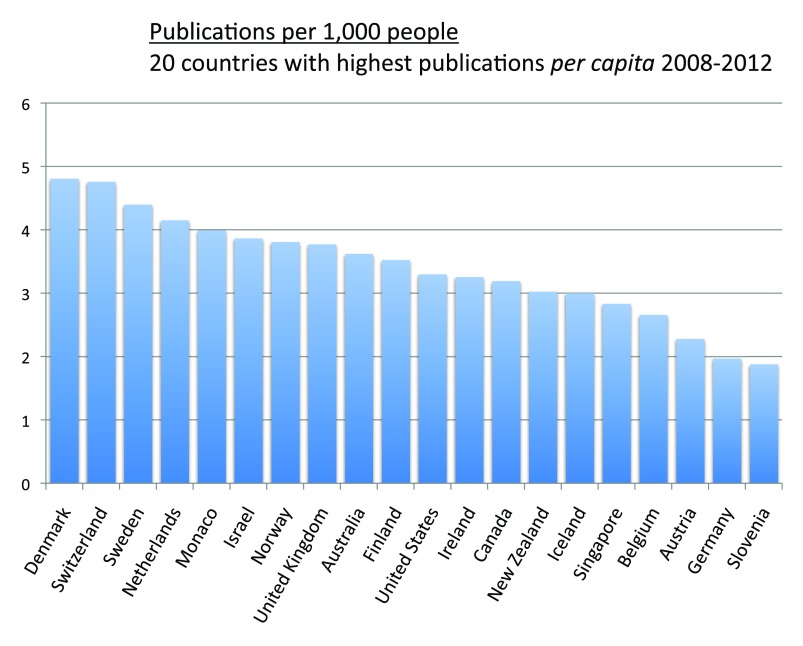
Publications (reviews excluded) *per capita* in the 20 countries with the highest publication (reviews excluded) *per capita* ratios in the 5-year period 2008–2012. The numbers represent publications per 1,000 people and were obtained by dividing the number of publications (reviews excluded) by the country populations and multiplying by 1,000. Criteria A.

**Figure S6.**
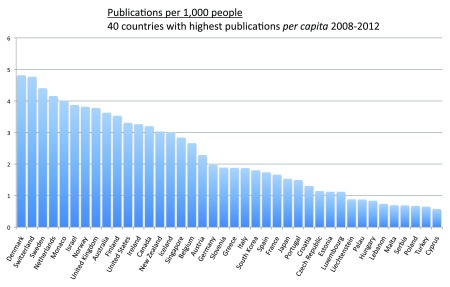
Publications (reviews excluded) *per capita* of the 40 countries with the highest publication (reviews excluded) *per capita* in the 5-year period 2008–2012. The numbers represent publications per 1,000 people and were obtained by dividing the number of publications (reviews excluded) by the country populations and by multiplying by 1,000. Criteria A.

We measured the publications *per capita* of countries (sheet 5). We divided the country publications in the time period 2008–2012 by the country population and multiplied by 1,000 in order to obtain the numbers of publication per 1,000 people. Figure 5 shows the publications per 1,000 people for the 20 countries with the most publications. Switzerland (4.8), Sweden (4.4), the Netherlands (4.2), Israel (3.9), the United Kingdom (3.8), and Australia (3.6) were, in descending order, the countries with the highest publications *per capita*. Iran (0.4), Brazil (0.3), China (0.2), and India (less than 0.1) were, in descending order, the countries with lowest publications *per capita*. We also ranked all other countries based on their publications *per capita*. Figure 6 and Figure S6 show the number of publications per 1,000 people for the 20 and the 40 countries with the highest publications *per capita*, respectively. The top ranked country was Denmark (which is not part of the group of 20 countries with the most publications) with 4.8 publications per 1,000 people in the time period 2008–2012. Switzerland (4.8), Sweden (4.4), and the Netherlands (4.2) closely followed Denmark in this ranking.

### Among the 40 countries with the most publications, Israel has the highest publications per GDP

**Figure 7.**
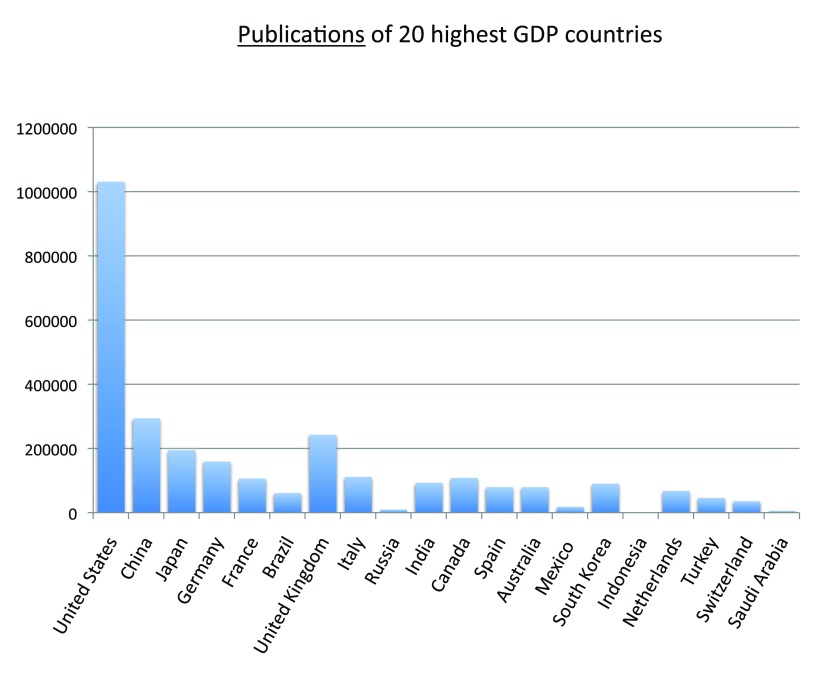
Numbers of publications (reviews excluded) in the 20 countries with the highest gross domestic products (GDP) in the 5-year period 2008–2012. Criteria A.

**Figure 8.**
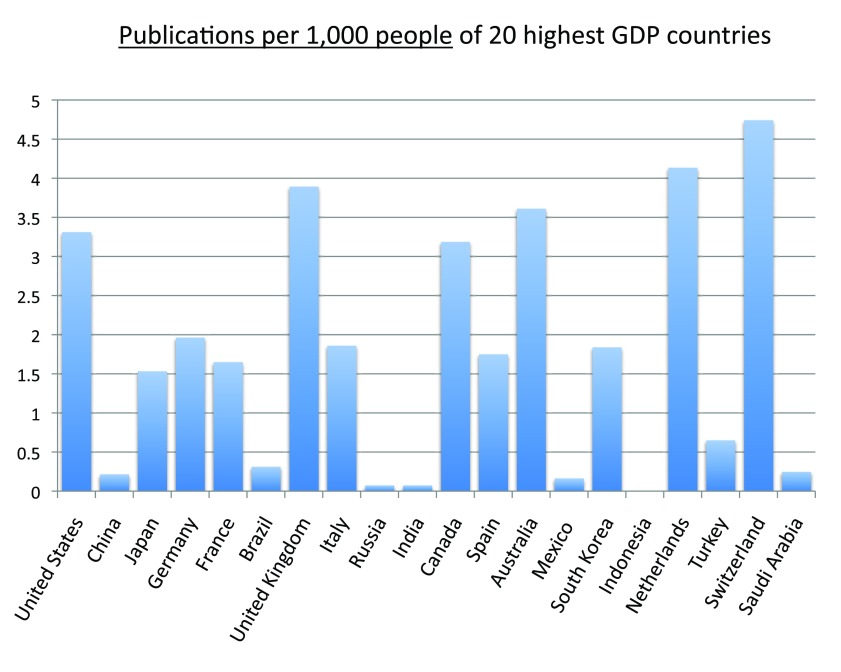
Publications (reviews excluded) *per capita* in the 20 countries with the highest gross domestic products (GDP) in the 5-year period 2008–2012. The numbers represent publications per 1,000 people and were obtained by dividing the number of publications (reviews excluded) by the country population and multiplying by 1,000. Criteria A.

**Figure S7.**
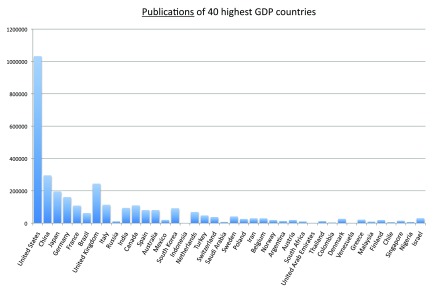
Publications (reviews excluded) of the 40 countries with the highest GDP in the 5-year period 2008–2012. Criteria A.

We analyzed the publication output of the 40 countries with the highest GDPs for the year 2011 and reported the data for the 20 and 40 countries with the highest GDPs, respectively in Figure 7 and Figure S7 (sheet 6). Almost every country in this group is also in the group of countries with the most publications in the time period 2008–2012; the exceptions are Russia, Mexico, Indonesia, and Saudi Arabia, which are not part of the group with the most publications, and Sweden, Israel, Iran, and Belgium, which are not in the group of 20 countries with the highest GDPs. We also calculated the publications per 1,000 people for the 20 countries with the highest GDPs: Switzerland (4.8), the Netherlands (4.2), and the United Kingdom (3.8), in descending order, had the highest ratios, whereas China (0.2), Russia (less than 0.1), India (less than 0.1), and Indonesia (less than 0.1), in descending order, had the lowest ratios in the time period 2008–2012 ( Figure 8) (sheet 6).

**Figure 9.**
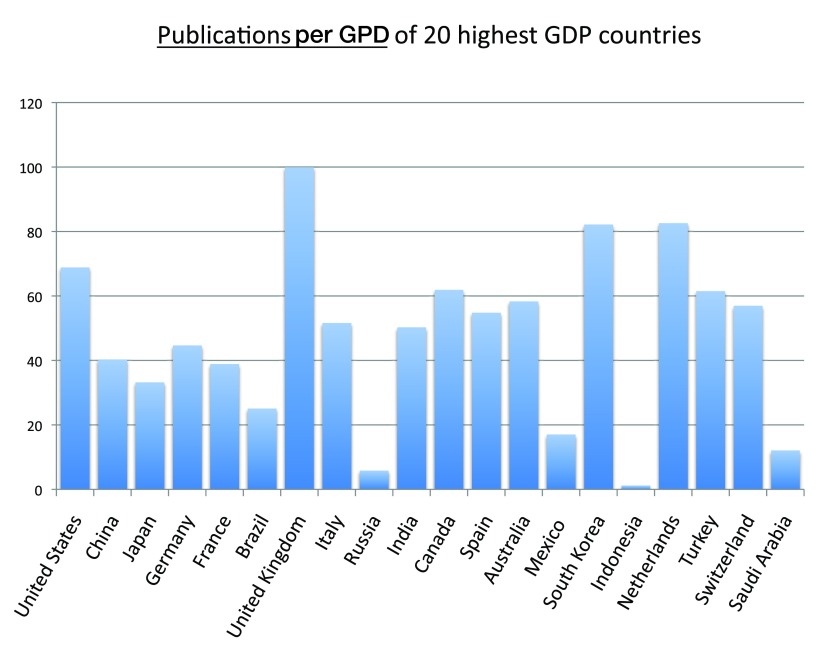
Publications (reviews excluded)-to-GDP ratios in the 20 countries with the highest GDP in the 5-year period 2008–2012. The numbers of publications (reviews excluded) were divided by the GDPs of 2011 (US$) from World Bank database and multiplied by 10 ^9^. Criteria A.

**Figure S8.**
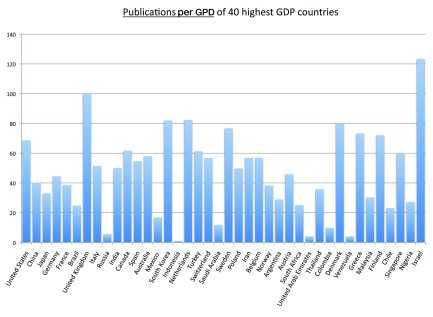
Publications (reviews excluded)-to-GDP ratios in the 40 countries with the highest GDP in the 5-year period 2008–2012. The numbers of publications (reviews excluded) were divided by the GDP of 2011 (US$) from World Bank database and multiplied by 10 ^9^. Criteria A.

Moreover, we calculated the number of publications per GDP for the 20 and 40 countries with the highest GDPs ( Figure 9 and Figure S8) (sheet 6). In the group of 40 countries with the highest GDPs, the country with the highest publications-to-GDP ratio in the time period 2008–2012 was Israel, followed by the United Kingdom, the Netherlands, and South Korea whereas Russia, the United Arab Emirates, Venezuela, and Indonesia were, in descending order, the countries with the lowest ratios. The ratio of the lowest ranking country (Indonesia) was less than 1% of the ratio of the highest ranking country (Israel).

### Among the 20 countries with the most publications, the United Kingdom has the highest publications per R&D (research and development) expenditure

**Figure 10.**
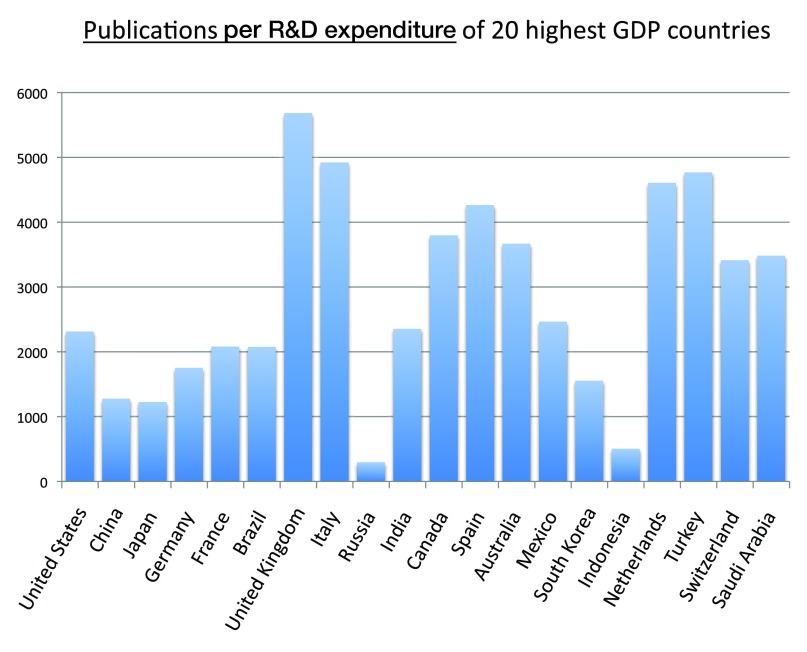
Publications (reviews excluded) per R&D expenditure of the 20 countries with the highest GDP in the 5-year period 2008–2012. The numbers of publications (reviews excluded) were divided by the R&D expenditure of 2012 expressed as GERD (Gross Expenditures on Research and Development) in billion US$ at Purchasing Power Parity (PPP). The data of R&D expenditures of countries were taken from the Battelle nonprofit private company ( http://www.battelle.org/docs/tpp/2014_global_rd_funding_forecast.pdf). Criteria A.

We also calculated the publications per R&D expenditure for the 20 countries with the highest GDPs. The numbers on the chart represent the ratios between the numbers of publications and these R&D expenditures. The United Kingdom, Italy, Turkey and the Netherlands were, in descending order, the countries with the highest ratios, whereas Indonesia and Russia were, in descending order, the countries with the lowest ratios in the time period 2008–2012 ( Figure 10) (sheet 9). The ratio of the lowest ranking country (Russia) is 5.1% of the ratio of the highest ranking country (the United Kingdom).

### The United States has the most publications of Clinical Trial studies

**Figure 11.**
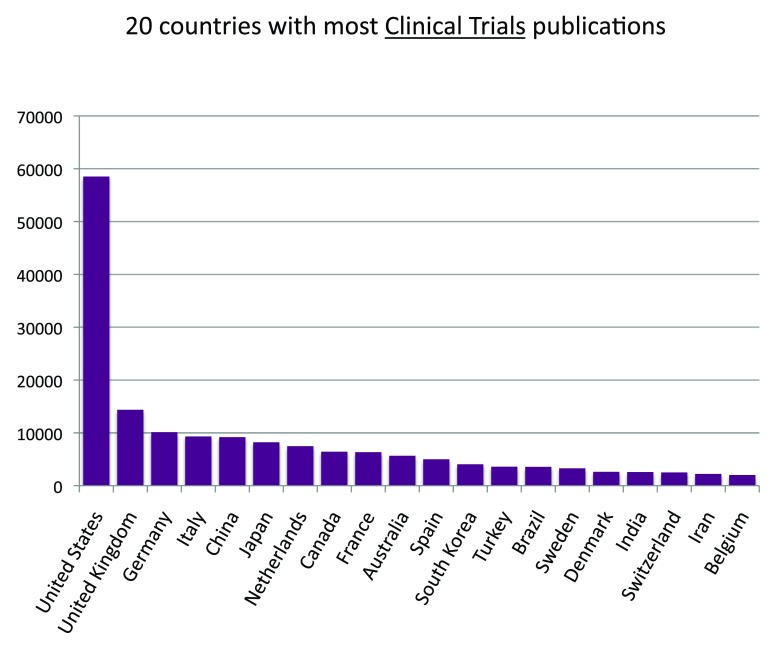
Numbers of Clinical Trial publications (reviews excluded) in the 20 countries with the most Clinical Trial publications (reviews excluded) in the 5-year period 2008–2012. Criteria E.

**Figure 12.**
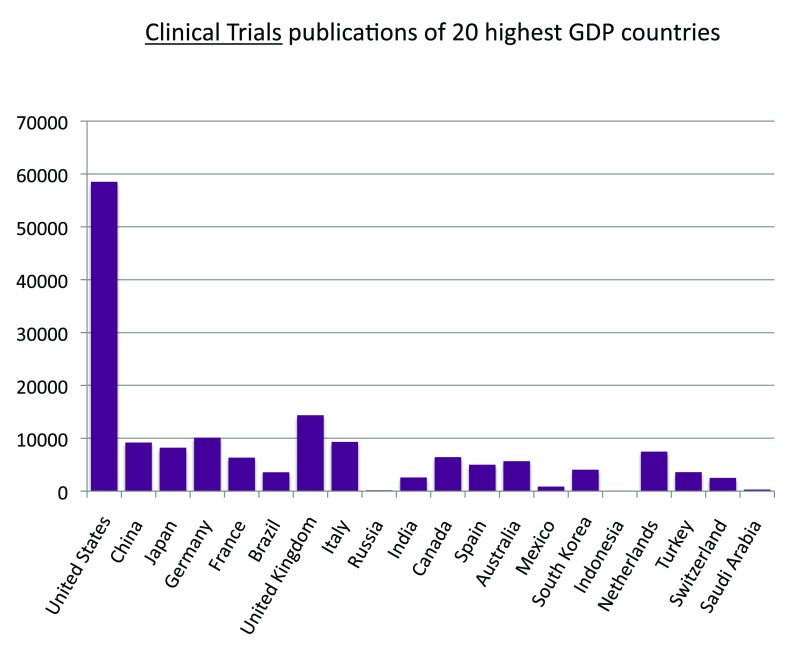
Numbers of Clinical Trial publications (reviews excluded) in the 20 countries with the highest GDP in the 5-year period 2008–2012. Criteria E.

**Figure S9.**
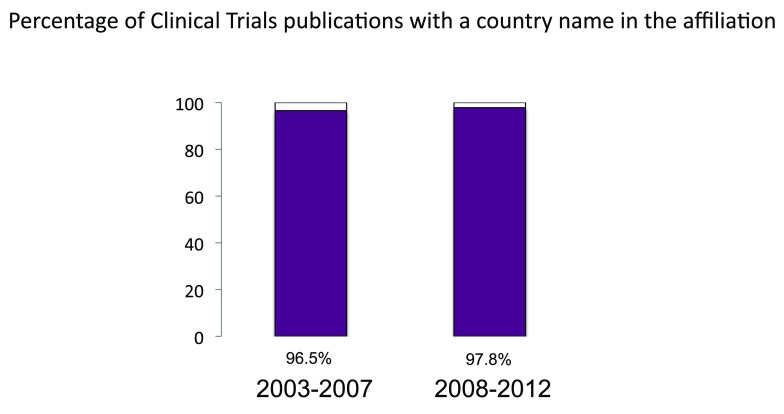
Percentages of Clinical Trial publications (reviews excluded) with country name in the affiliation field. Criteria E were used to calculate the proportions in the 5-year periods 2003–2007 and 2008–2012. The total world Clinical Trials publications (reviews excluded) were obtained by searches without any specification in the affiliation field. The total world Clinical Trials publications (reviews excluded) with the country name in the affiliation field were obtained by summing the numbers of Clinical Trials publications (reviews excluded) in all countries of the world.

We measured the publication output based on clinical trial studies. First, similarly to what we did with the general publications ( Figure S1), we calculated the proportions of clinical trials publications with a country name in the affiliation. The proportions were 96.5% and 97.8% for the time periods 2003–2007 and 2008–2012, respectively ( Figure S9) (sheet 10). In the time period 2008–2012, there were over 180,000 publications based on clinical trial studies (sheet 10). We then determined the numbers of clinical trial publications for all the countries of the world (sheet 10) and ranked the 20 countries with the most clinical trial publications ( Figure 11) (sheet 10) in the time period 2008–2012. The United States was the country with the most publications of clinical trial studies, with over 58,000 publications in the time period 2008–2012, over four times the volume of the United Kingdom (with over 14,000 clinical trial publications), the second in the ranking. In Figure 12 (sheet 11), we show the clinical trial publications of the 20 countries with the highest GDPs.

### Among the 20 countries with the most publications, Iran has the highest increase of publications over the last 10 year

**Figure 13.**
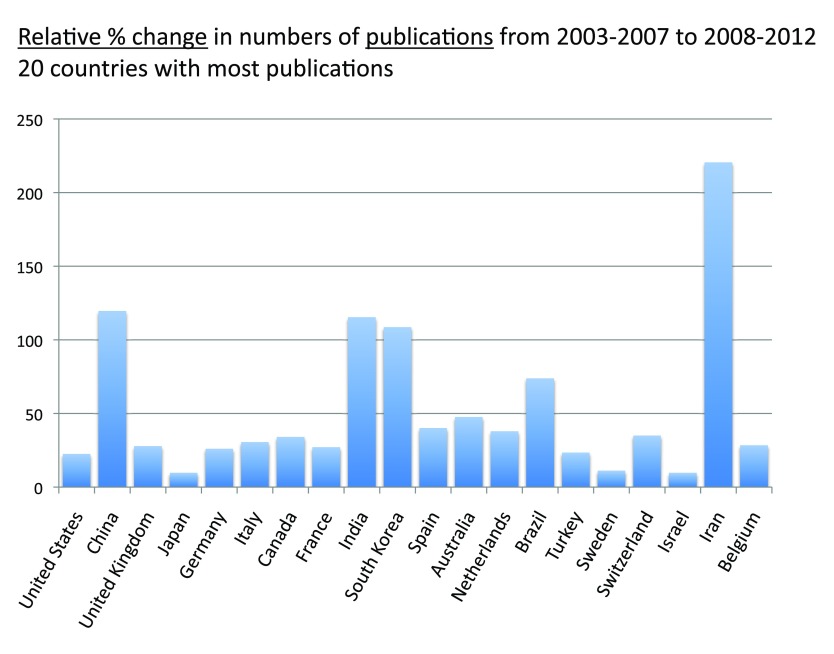
Relative changes in numbers of publications (reviews excluded) from the 5-year period 2003–2007 to the 5-year period 2008–2012 for the 20 countries with the most publications (reviews excluded). The relative changes are expressed as percentage change relative to the 5-year period 2003–2007. Criteria A.

**Figure 14.**
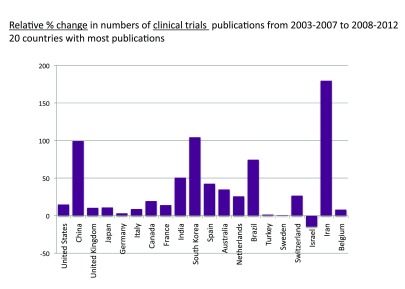
Relative changes in numbers of Clinical Trial publications (reviews excluded) from the 5-year period 2003–2007 to the 5-year period 2008–2012 for the 20 countries with the most publications (reviews excluded). The relative changes are expressed as percentage change relative to the 5-year period 2003–2007. Criteria E.

We compared the volumes of publications of time periods 2003–2007 and 2008–2012 for the 20 countries with the most publications. Figure 13 (sheet 12) shows the relative change (as the percentage of the volume of time period 2003–2007) of general publications (as usual, reviews were excluded). The four countries with the highest increases were, in descending order, Iran (220.4%), China (119.5%), India (115.2%), and South Korea (108.6%); these countries more than doubled the volume of publications from time period 2003–2007 to time period 2008–2012. The volumes of publications did not decrease in any of the 20 countries with the most publications. In this group the country with the lowest increase was Japan, with a 9.7% increase. We also determined the relative changes with regard to the clinical trial publications. Figure 14 (sheet 13) shows these relative changes. The country with the highest relative change was Iran, with a 179.6% increase from time period 2003–2007 to time period 2008–2012. South Korea (104.3%) and China (99.3%) were second and third in this ranking. The volume of clinical trial publications decreased only for Israel (-14.6%).

### Clinical Trial Attraction Scores

**Figure 15.**
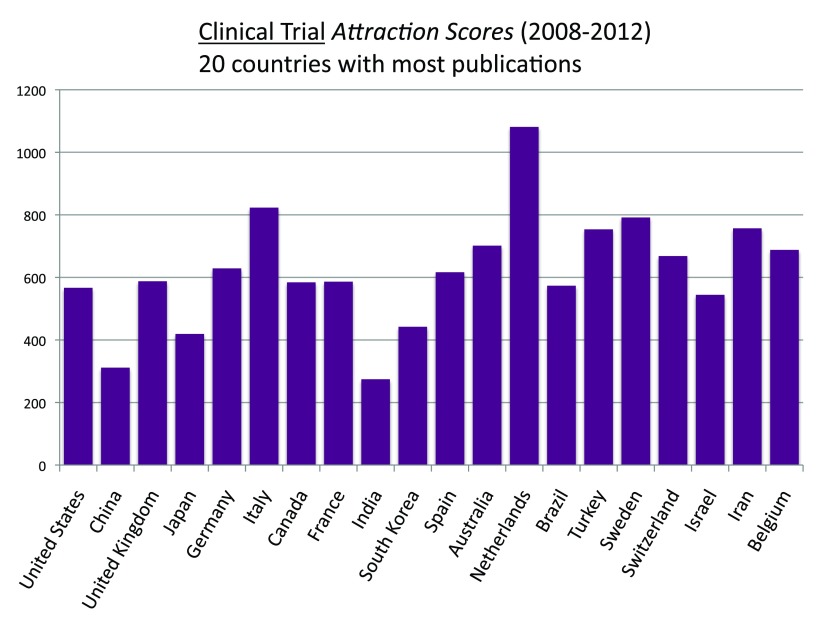
“Clinical Trial Attraction Scores” for the 20 countries with the most publications (reviews excluded) in the 5-year period 2008–2012. The “Clinical Trial Attraction Scores” were calculated by dividing the numbers of Clinical Trials publications (reviews excluded) by the total numbers of publications (reviews excluded) in the 5-year period 2008–2012 and multiplying by 10,000. Criteria A and E.

In order to determine the proportion of publications that are clinical trial studies, a proxy for the level of “attractiveness” towards clinical trial investigations, we created the Clinical Trial Attraction Score, defined as the ratio of clinical trial publications to the general publications multiplied by 10,000 (to make these scores comparable to the Topic Attraction Scores, see below). We calculated these scores for the 20 countries with the most publications ( Figure 15) (sheet 14). The Netherlands, Italy, and Sweden were, in descending order, the countries with the highest Clinical Trial Attraction Scores. China and India, in descending order, were the ones with the lowest. The Attraction Score of the highest ranking country (the Netherlands) was 3.9 times the Attraction Score of the lowest ranking country (India).

### Area/Topic Attraction Scores

As a proxy for the level of “attractiveness” towards specific research areas or topics, we determined Attraction Scores for research areas or topics. We used the same method used to determine the Clinical Trial Attraction Score. Basically, the number of publications related to a specific area or topic was divided by the total number of publications of the same country and then multiplied by 10,000 (to get easily readable scores).

**Figure 16.**
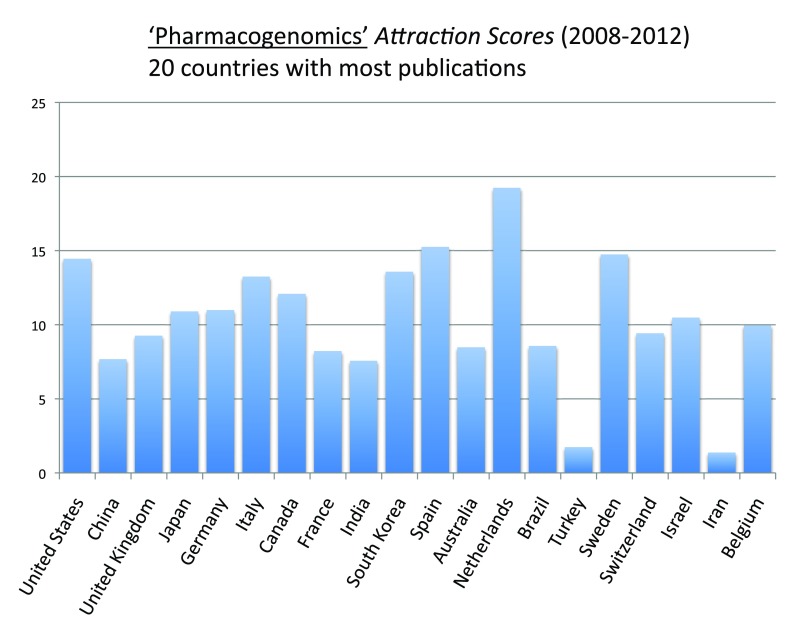
“Pharmacogenomic Attraction Scores” for the 20 countries with the most publications (reviews excluded) in the 5-years period 2008–2012. The “Topic Attraction Scores” were calculated by dividing the numbers of publications (reviews excluded) with “pharmacogenomic” OR “pharmacogenomics” in the title/abstract field by the total numbers of publications (reviews excluded) in the 5-year period 2008–2012 and multiplying by 10,000. Criteria B.

Figure 16 (sheet 15) reports the Topic Attraction Scores for “pharmacogenomics” in the 20 countries with the most publications in the time period 2008–2012. The Netherlands, Spain, Sweden, and the United States were, in descending order, the countries with the highest scores while Turkey and Iran were, in descending order, the countries with the lowest scores. The Attraction Score of the highest ranking country (the Netherlands) was 14.1 times the Attraction Score of the lowest ranking country (Iran).

**Figure 17.**
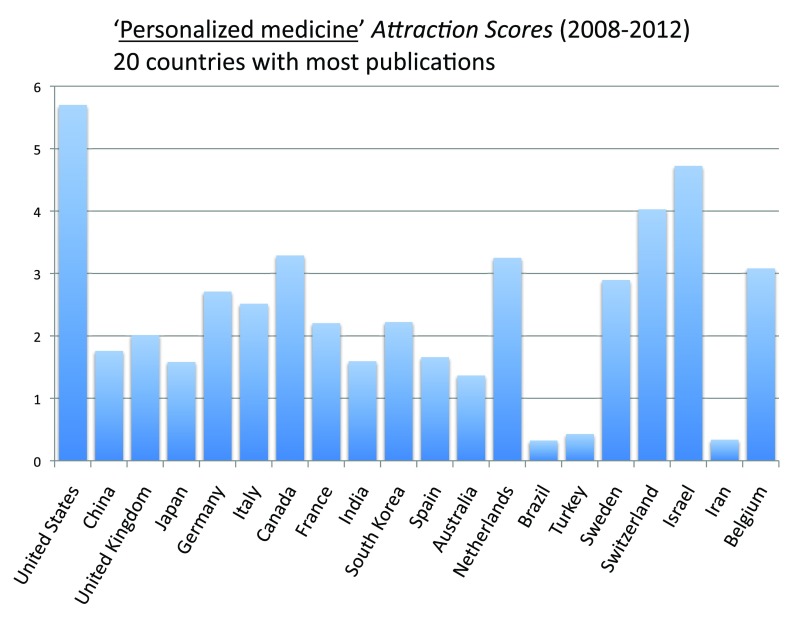
“Personalized Medicine Attraction Scores” for the 20 countries with the most publications (reviews excluded) in the 5-year period 2008–2012. The “Topic Attraction Scores” were calculated by dividing the numbers of publications (reviews excluded) with “personalized medicine” in the title/abstract field by the total numbers of publications (reviews excluded) in the 5-year period 2008–2012 and multiplying by 10,000. Criteria B.

Figure 17 (sheet 16) reports the Topic Attraction Scores for “personalized medicine” of the 20 countries with the most publications in the time period 2008–2012. United States, Israel, and Switzerland were, in descending order, the countries with the highest scores while Turkey, Iran and Brazil were, in descending order, the ones with the lowest scores. The Attraction Score of the highest ranking country (the United States) was 17.3 times the Attraction Score of the lowest ranking country (Brazil).

**Figure 18.**
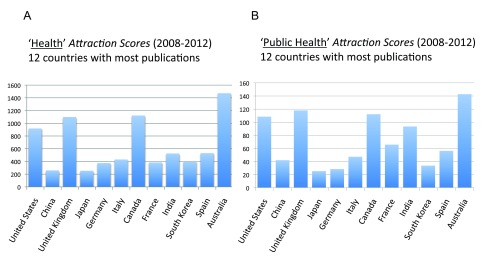
“Health Attraction Scores” and “Public Health Attraction Scores” for the 12 countries with the most publications (reviews excluded) in the 5-year period 2008–2012. ( **A**) The “Topic Attraction Scores” were calculated by dividing the numbers of publications (reviews excluded) with “health” in the title/abstract field by the total numbers of publications (reviews excluded) in the 5-year period 2008–2012 and multiplying by 10,000. ( **B**) The “Topic Attraction Scores” were calculated by dividing the numbers of publications (reviews excluded) with “public health” in the title/abstract field by the total numbers of publications (reviews excluded) in the 5-year period 2008–2012 and multiplying the obtained quotient by 10,000. Criteria B.

Figure 18 (sheet 17) reports the Topic Attractions Scores for “health” and “public health” for the 12 countries with the most publications in the time period 2008–2012. Australia, the United Kingdom, Canada, and the United States (remarkably, all Anglo-Saxon countries) had, in descending order, the highest Public Health Attraction Scores (with very similar results for the Health Attraction Score). The Attraction Score of the highest ranking country (Australia) was 5.6 times the Attraction Score of the lowest ranking country (Japan).

**Figure 19.**
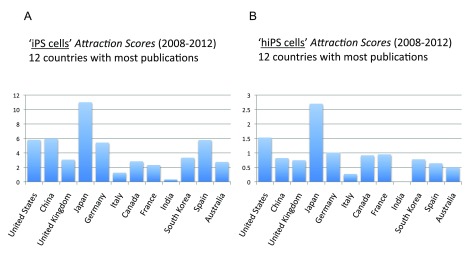
“iPS cells Attraction Scores” and “hiPS cells Attraction Scores” for the 12 countries with the most publications (reviews excluded) in the 5-year period 2008–2012. ( **A**) The “Topic Attraction Scores” were calculated by dividing the numbers of publications (reviews excluded) with “induced pluripotent stem cells” in the title/abstract field by the total numbers of publications (reviews excluded) in the 5-year period 2008–2012 and multiplying by 10,000. ( **B**) The “Topic Attraction Scores” were calculated by dividing the numbers of publications (reviews excluded) with “human induced pluripotent stem cells” in the title/abstract field by the total numbers of publications (reviews excluded) in the 5-years period 2008–2012 and multiplying by 10,000. Criteria B.

Figure 19 (sheet 18) reports the Topic Attraction Scores for “induced pluripotent stem cells” and “human induced pluripotent stem cells” of the 12 countries with the most publications in the time period 2008–2012. In both cases Japan was the country with by far the highest score. The Attraction Score for “iPS cells” of the highest ranking country (Japan) was 34.4 times the Attraction Score of the lowest ranking country (India). The Attraction Score for “hiPS cells” of the lowest ranking country (India) was 0.

**Figure 20.**
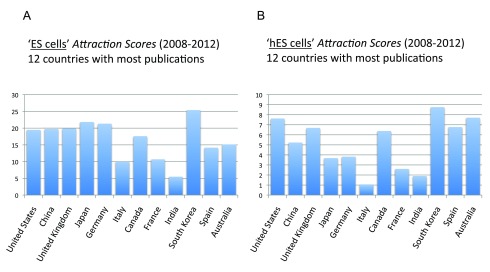
“ES cells Attraction Scores” and “hES cells Attraction Scores” for the 12 countries with the most publications (reviews excluded) in the 5-year period 2008–2012. ( **A**) The “Topic Attraction Scores” were calculated by dividing the numbers of publications (reviews excluded) with “embryonic stem cells” in the title/abstract field by the total numbers of publications (reviews excluded) in the 5-year period 2008–2012 and multiplying by 10,000. ( **B**) The “Topic Attraction Scores” were calculated by dividing the numbers of publications (reviews excluded) with “human embryonic stem cells” in the title/abstract field by the total numbers of publications (reviews excluded) in the 5-year period 2008–2012 and multiplying by 10,000. Criteria B.

Figure 20 (sheet 19) reports the Topic Attraction Scores for “embryonic stem cells” and “human embryonic stem cells” in the 12 countries with the most publications in the time period 2008–2012. In both cases, South Korea was the country with the highest score. The Attraction Score for “ES cells” of the highest ranking country (South Korea) was 4.6 times the Attraction Score of the lowest ranking country (India). The Attraction Score for “hES cells” of the highest ranking country (South Korea) was 8.1 times the Attraction Score of the lowest ranking country (Italy).

### A relative comparison with all-disciplines’ citable documents

**Figure S10.**
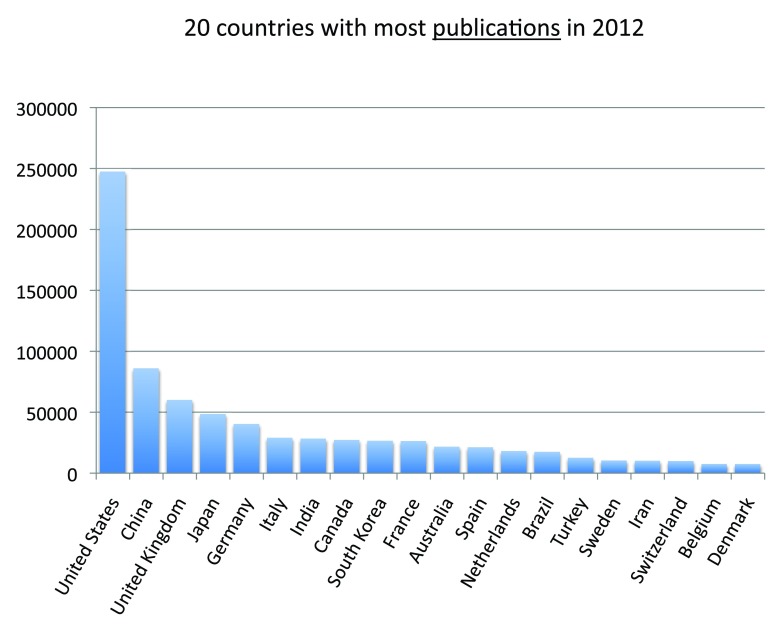
Numbers of publications (reviews excluded) of the 20 countries with the most publications (reviews excluded) in the year 2012. Criteria A.

**Figure S11.**
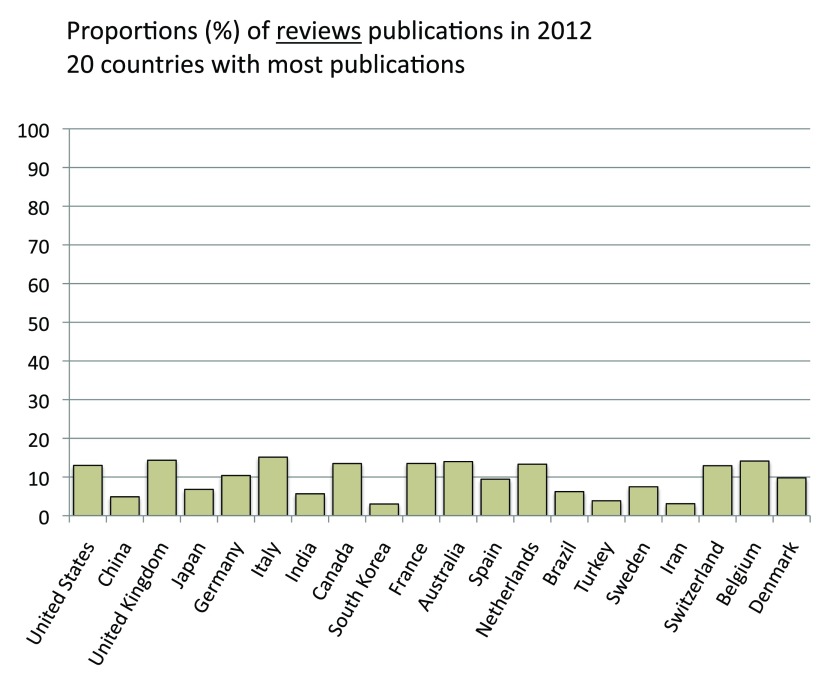
Estimation of the percentages of review publications of the 20 countries with the most publications (reviews excluded) in the year 2012. The percentages were calculated by determining the proportions between the numbers of publications obtained with Criteria A and the numbers of publications obtained with Criteria A but without the exclusion of the reviews.

**Figure S12.**
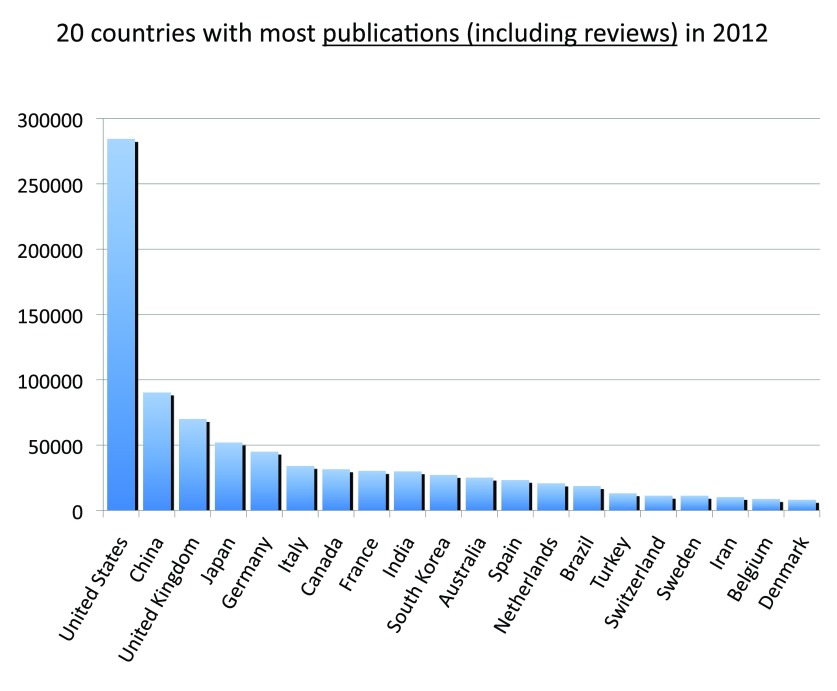
Numbers of publications (reviews included) of the 20 countries with the most publications in the year 2012. Criteria A (without reviews exclusion).

**Figure S13.**
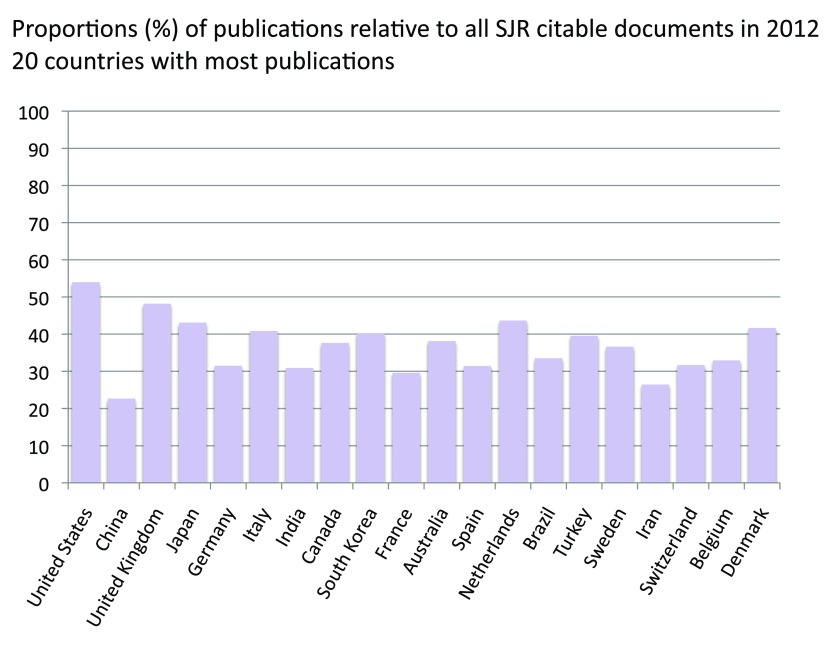
Proportions between the numbers of publications (reviews included) and the SJR citable documents of the 20 countries with the most publications in the year 2012. The proportions were calculated by dividing the numbers of publications (reviews included) and all the citable documents of the SJR database (SCImago Journal & Country Rank) of the 20 countries with the most publications in the year 2012.

We compared publication output in the life sciences with the overall output of all disciplines. Data on the latter were obtained from the SCImago Journal & Country Rank, which includes journals and country scientific indicators from the information contained in the Scopus® database ( http://www.scimagojr.com/). We ran the comparison for a single year (2012). Figure S10 (sheet 21) reports data for the 20 countries with the most publications in year 2012. The pattern, as expected, was very similar to the pattern for the time period 2008–2012 ( Figure 3), and the group of 20 countries was the same with the exception of Israel, which was only in the 2008–2012 group and Denmark, which was only in the 2012 group. In addition, this time we also included reviews as these are included in the data from SCImago Journal & Country Rank. Figure S11 (sheet 21) reports the percentage of reviews (estimated as previously indicated) of the 20 countries with the most publications in 2012. With an average of 9.7%, a minimum of 3.0% (South Korea) and a maximum of 15.1% (Italy), these results are similar to those from 2008–2012 ( Figure S5). We then included reviews and re-ranked the same 20 countries shown in Figure S10 ( Figure S12) (sheet 21) and found that, given that the percentage of reviews was low, rankings and relative differences are not substantially affected by the inclusion of reviews. We therefore estimated the ratio of publications in the life sciences with publications in all fields and reported the results in Figure S13 (sheet 21). Data show an average of 36.7% publications in the life sciences with China (22.7%) and Iran (26.4%) scoring at the bottom and the United States (54.0%) and the United Kingdom (48.2%) scoring at the top.

### Most of the publications in English are produced in countries where English is not the primary language

**Figure 21.**
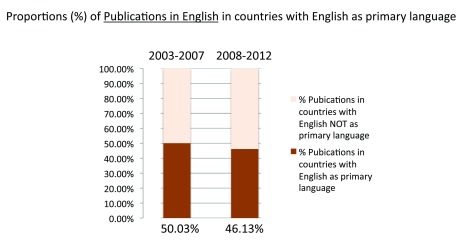
Proportion of publications (reviews excluded) written in English in countries where English is the primary language in the 5-year periods 2003–2007 and 2008–2012. The proportions are expressed as percentages in the 5-year periods 2003–2007 and 2008–2012. The proportions were determined by dividing the sum of the numbers of publications (reviews excluded) in all the countries where English is the primary language by the sums of the numbers of publications (reviews excluded) in all countries of the world. For countries that we considered to have English as primary language see the data file (sheet 21). Criteria B.

We calculated the numbers of publications of countries where English is or is not the primary language. Figure 21 (sheet 22) shows the proportions of publications from countries where English is the primary language for the two time periods 2003–2007 and 2008–2012. The percentage of publications of these countries slightly decreased from 50.0% in time period 2003–2007 to 46.1% in time period 2008–2012. As a consequence, the percentages of publications of countries where English is not the primary language increased from 50.0% to 53.9%. This increase was expected given the large increases in publications volumes in non-English native speaking, high-volume publishing countries like China, India, South Korea, and Iran ( Figure 13). For a list of countries considered to have English as primary language, see the data file.

### Nearly 70% of publications are conceived in North-Central America or Europe

**Figure 22.**
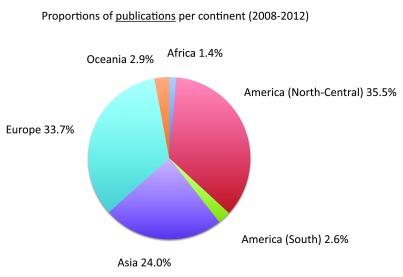
Proportions of publications (reviews excluded) per continent in the 5-year period 2008–2012. The proportions are expressed as percentages. America was divided into North-Central America and South America. The numbers of publications (reviews excluded) for Asia and Europe were approximated by equally dividing the publications (reviews excluded) of Russia and Turkey between the two continents. Criteria B.

**Figure 23.**
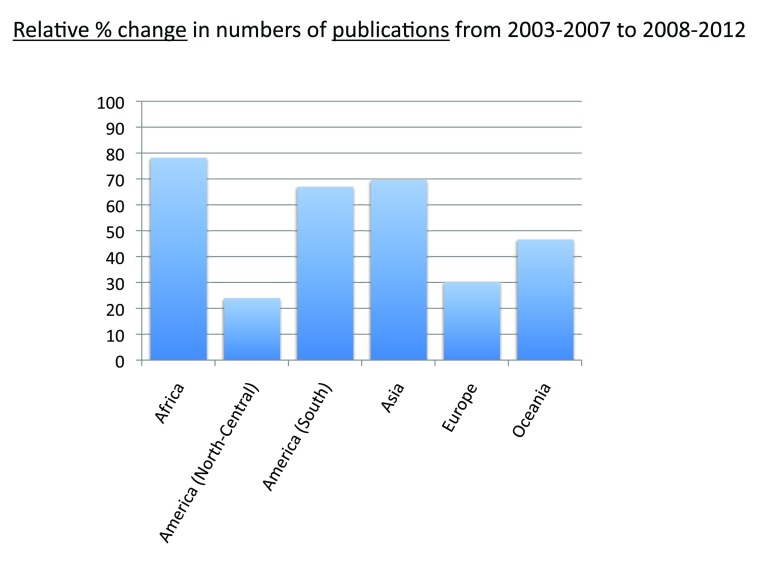
Relative changes in numbers of publications (reviews excluded) by continent from the 5-year period 2003–2007 to the 5-year period 2008–2012. The relative changes are expressed as percentages relative to the number of publications (reviews excluded) of the 5-year period 2003–2007. Criteria B.

**Figure 24.**
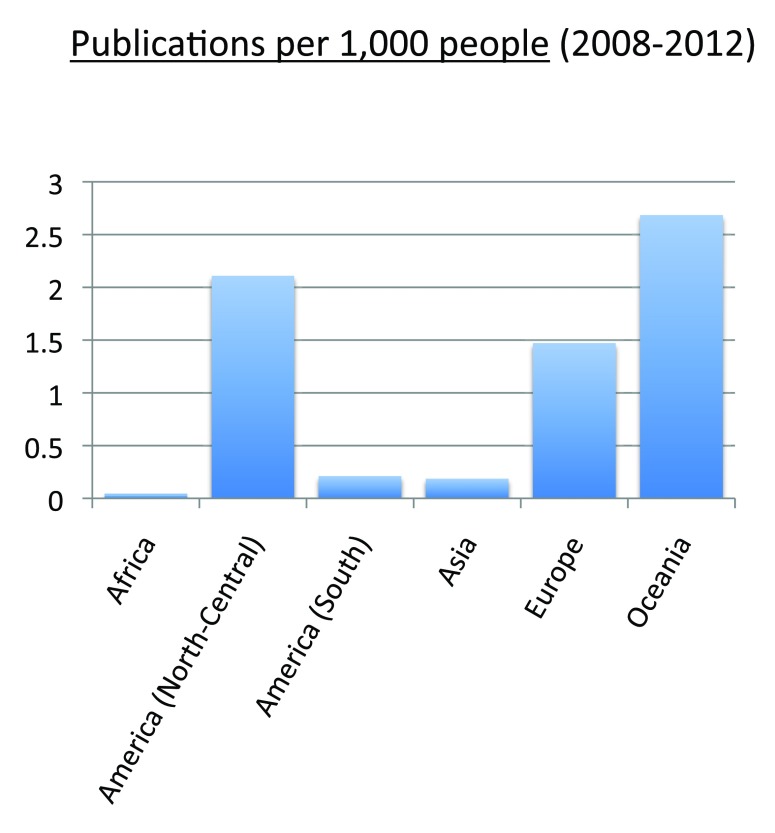
Publications (reviews excluded) *per capita* by continent in the 5-year period 2008–2012. The numbers represent the numbers of publications (reviews excluded) per 1,000 people by continent in the 5-year period 2008–2012. Criteria B.

Data on publication output in each continent in the time period 2008–2012 appear in Figure 22 (Sheet 23). To calculate publication output for each continent, we divided the American continent into North-Central America and South America. North-Central America and Europe were the continents with the greatest number of publications, representing 35.5% and 33.7% of the total numbers of publications (sum of all the countries), respectively. Figure 23 shows the relative changes in publications from time period 2003–2007 to time period 2008–2012. Africa was the continent with the biggest relative change in publications, with a 78.5% increase in publication volume from time period 2003–2007 to time period 2008–2012, whereas Europe (30.4%) and North-Central America (24.2%) were the continents with the smallest increases. We also calculated the publications *per capita* in the time period 2008–2012. Figure 24 (sheet 23) reports the publications per 1,000 people in the continents. Oceania was the continent with the highest publications *per capita* with 2.7 publications per 1,000 people, followed by North-Central America with 2.1 and Europe with 1.5. Africa was the continent with the smallest publications *per capita*, with less than 0.1 publications per 1,000 people.

### Proportions of publications of the European Union

**Figure S14.**
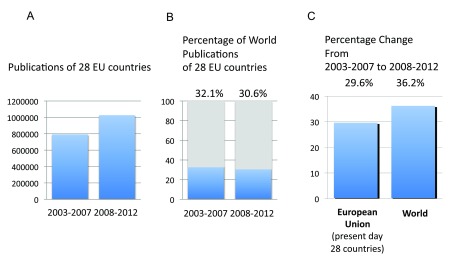
Publications of the European Union. ( **A**) Numbers of publications (reviews excluded) of the present day 28 countries of the European Union in the 5-year periods 2003–2007 and 2008–2012. ( **B**) Share of total world publications (reviews excluded) that came from the present day 28 countries of the European Union in the 5-year periods 2003–2007 and 2008–2012. ( **C**) Relative changes in numbers of publications (reviews excluded) of the present day 28 countries of the European Union and of the World from the 5-year period 2003–2007 to the 5-year period 2008–2012. The changes are expressed as percentage changes relative to the 5-year period 2003–2007. Criteria A.

**Figure S15.**
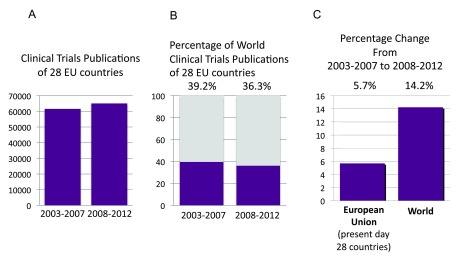
Clinical Trial Publications of the European Union. ( **A**) Numbers of Clinical Trials publications (reviews excluded) of the present day 28 countries of the European Union in the 5-year periods 2003–2007 and 2008–2012. ( **B**) Share of total world Clinical Trials publications (reviews excluded) that came from the present day 28 countries of the European Union in the 5-year periods 2003–2007 and 2008–2012. ( **C**) Relative changes in numbers of Clinical Trial publications (reviews excluded) of the present day 28 countries of the European Union and of the World from the 5-year period 2003–2007 to the 5-year period 2008–2012. The changes are expressed as percentage changes relative to the 5-year period 2003–2007. Criteria A and E.

We calculated the number of publications of the 28 countries that are currently part of the European Union (EU Countries) in time periods 2003–2007 and 2008–2012. The publications during the time period 2008–2012 were more than one million ( Figure S14A) (sheet 24) and represented 32.1% and 30.6% of the world’s total publications in the time periods 2003–2007 and 2008–2012, respectively ( Figure S14B). The relative increase from time period 2003–2007 to time period 2008–2012 of the EU Countries was 29.6%, therefore smaller than the relative increase for the whole world (36.2%) ( Figure S14C). We also calculated the total clinical trials study publications of the EU Countries in time periods 2003–2007 and 2008–2012 ( Figure S15A). In time period 2008–2012, there were over 65,000 clinical trial publications. These publications represent 39.2% and 36.3% of the world’s total clinical trials publications in time periods 2003–2007 and 2008–2012, respectively ( Figure S15B). The relative change in clinical trial publications from time period 2003–2007 to time period 2008–2012 was a 5.7% increase for the 28 countries, compared to a 14.2% increase for the whole world ( Figure S15C).

### Comparison between custom range and the automatic annual counts

**Figure S16.**
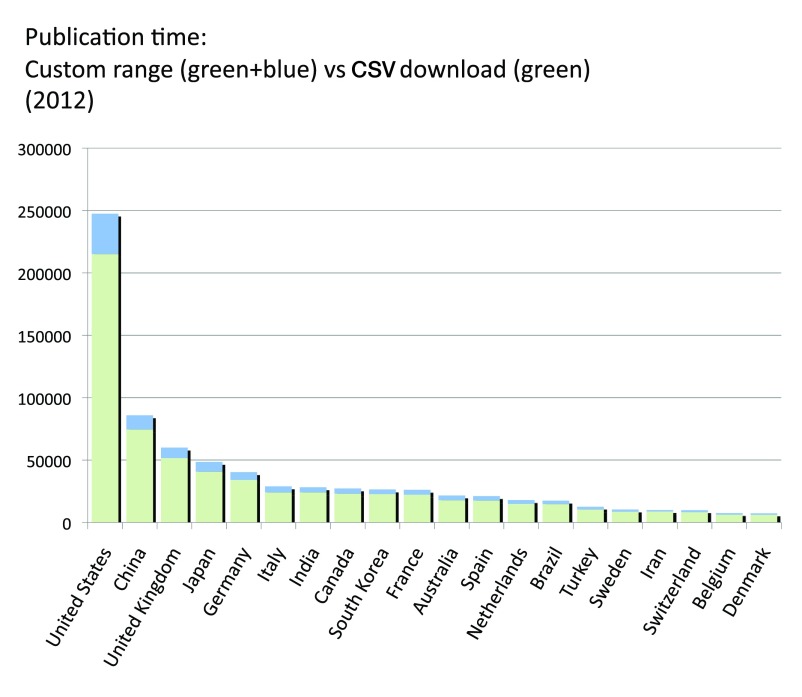
A comparison between automatic CSV downloads and custom range searches. Numbers of publications (reviews excluded) of the 20 countries with the most publications (reviews excluded) in the year 2012 are shown, either obtained by automatic CSV downloads (pale green) or by custom range searches (pale green + pale blue). The pale blues portions of the bars represent the differences between the two methods. Criteria A.

PubMed provides a function to automatically download CSV files with the numbers of publications year by year. We compared the numbers of publications obtained by using this function with the numbers of publications obtained by using the custom range function to define time periods that we described above. An analysis of the 2012 reveals that the CSV download yielded fewer publications than the custom range approach. Figure S16 (sheet 25) shows the differences for the 20 countries with the most publications in 2012. On average the difference was -18.4%, with a maximum difference of -21.7% for Turkey and a minimum difference of -13.8% for Iran.

### Update: The patterns of countries’ publications in 2013 and 2014 are similar

We determined the numbers of publications of the 20 countries with the most publications for the years 2013 and 2014. We observed that the patterns of countries' publications were similar in the two years. We also analyzed as before the review publications and noticed that the patterns were similar ( Figure S18– Figure S25).

## Discussion

### A methodology for in-depth assessment of bio-publication output of countries

Assessing the quantity and quality of a country’s scientific output is key to understanding and improving its research system. In this paper, we present a methodology that focuses on publication in biology and related disciplines that contribute to advancing that field. Based on the search engine PubMed, the method was used to count publications at the country level and at the supranational level (continents and European Union), in different time periods, to trace changes in publication outcome over time and to measure publication ratios relative to country populations, country GDP and R&D expenditure, the share of publications based on clinical trials, whether the publication was a review article, and whether the publication was written in English. We demonstrated how various stringency criteria could be deployed to check data robustness. Box 1 presents an excerpt of the main findings.

Box 1.

1. The total number of publications during years 2008–2012 (almost four million, excluding review publications) is nearly the double the number of publications during years 1993–1997
2. The proportion of publications based on clinical trials has remained constant around 5% since 1993
3. The proportion of review publications for the 20 countries with the most publications is 9.9%, with a maximum for Italy (14.7%) and a minimum for South Korea (2.9%) (years 2008–2012)
4. The United States, with over one million publications in years 2008–2012 (reviews excluded) is by far the country with the most publications, having almost four times the publications of the second ranking country, China
5. The country with the most publications *per capita* is Denmark (4.81 per 1,000 people), followed by Switzerland (4.76 per 1,000 people) and Sweden (4.40 per 1,000 people) (years 2008–2012)
6. The country with the highest publications-to-GDP ratio, among the 40 countries with the most publications, is Israel, followed by the United Kingdom, the Netherlands, and South Korea (years 2008–2012)
7. The country with the highest publications-to-R&D expenditure (GERD PPP), among the 20 countries with the highest GDPs is the United Kingdom, followed by Italy, Turkey, and the Netherlands
8. Among the 20 countries with the most publications, Iran is the country with the highest relative increase (+220%) in the number of publications from years 2003–2007 to years 2008–2012. Japan is the country with the lowest relative increase (+9.7%)
9. Among the 20 countries with the most publications, Iran is the country with the highest relative increase (+179.6%) in the number of clinical trial publications from years 2003–2007 to years 2008–2012. Israel is the only country with a decrease (-14.6%)
10. The United States is the country with the most publications based on clinical trials (almost 60,000), nearly four times the number of publications based on clinical trials in the second ranking country, the United Kingdom (years 2008–2012)
11. Among the 20 countries with the most publications, the Netherlands is the country with the highest “Clinical Trial Attraction Score”, followed by Italy (years 2008–2012)
12. Among the 20 countries with the most publications, the Netherlands is the country with the highest “Pharmacogenomics Attraction Score” (years 2008–2012)
13. Among the 20 countries with the most publications, the United States is the country with the highest “Personalized Medicine Attraction Score” (years 2008–2012)
14. Among the 20 countries with the most publications, Japan is the country with the highest “iPS cells Attraction Score” (years 2008–2012)
15. Among the 20 countries with the most publications, South Korea is the country with the highest “hES cells Attraction Score”, (years 2008–2012)
16. There is a relationship between policies regulating the use of human embryonic stem cells and the “hES cells Attraction Scores”
17. Among the 12 countries with the most publications, Australia, the United Kingdom, and Canada, in descending order, have the highest “Public Health Attraction Scores” (years 2008–2012)
18. The estimated proportion of publications written in English in countries where English is the primary language has decreased from 50.0% (years 2003–2007) to 46.1% (years 2008–2012)
19. Oceania is the continent with the most publications *per capita* (2.7 per 1,000 people) followed by North-Central America (2.1 per 1,000 people) (years 2008–2012)
20. North-Central America has the highest proportion of world publications (35.5%); Africa has the lowest (1.4%) (years 2008–2012)
21. The proportion of world publications of the present day 28 countries of the European Union is 30.6% (years 2008–2012)

### Attraction Scores gauge the relative strength in certain types of research or on certain topics of inquiry

We have also created the Attraction Scores. These assess the focus of publication output on certain types of studies (e.g., clinical trials) or areas or topics of interest (e.g., hESC). Attraction Scores express a proportion of the total publication output rather than the number of papers. For this reason, we believe that they better represent the real attraction of certain types of studies or certain topics of research to scientists.

Attraction Scores can be correlated to many different factors. It is no surprise that this attraction for a cutting-edge topic like “pharmacogenomics” is higher in more technologically developed countries ( Figure 16). Higher Attraction Scores may also be determined by past path-breaking discoveries that attract investments and the attention of researchers. This seems to be the case of iPS cells and Japan, a country in which these cells were first obtained and then highly researched thanks to massive research and technological investment ^12– 14^. The predictable result is that Japan has indeed the highest Attraction Score for iPS cells of all surveyed countries. Variations in Attraction Scores can also be correlated with other factors, including the prevalence of certain diseases, the structure of research workforces (e.g. small groups vs. big collaborative efforts), regulatory policies, and cultural and political factors. Attraction Scores can be used not only to assess the effect of putative determining factors but also to predict future trends. For example, the focus toward certain areas (or topics) or types of research can have different consequences such as the development of new avenues of research or new types of interaction between academia and industry.

### Attraction Scores as a means to study research policy impact

**Figure S17.**
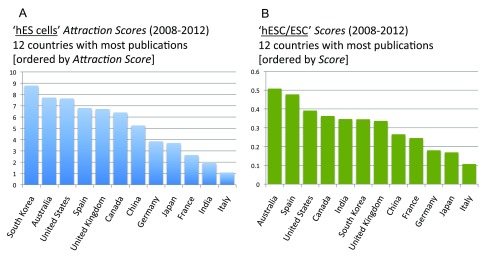
“hES cells Attraction Scores” and “hES cells/ES cells Scores” for the 12 countries with the most publications (reviews excluded) in the 5-year period 2008–2012. ( **A**) The “Topic Attraction Scores” were calculated by dividing the numbers of publications (reviews excluded) with “human embryonic stem cells” in the title/abstract field by the total numbers of publications (reviews excluded) in the 5-year period 2008–2012 and multiplying by 10,000. The countries are ranked according to the scores. ( **B**) The “hESC/ESC Scores” were calculated by dividing the numbers of publications (reviews excluded) with “human embryonic stem cells” in the title/abstract field by the numbers of publications (reviews excluded) with “embryonic stem cells” in the title/abstract field in the 5-year period 2008–2012 and multiplying by 10,000. The countries are ranked according to the scores. Criteria B.

Though a detailed and comprehensive analysis of correlations and predictive uses of the Attraction Scores is beyond the purposes of this study, we want to provide an example of how the Attraction Scores can be used to study the effect of specific policies. To this end, the policies regulating the use of hESCs in research present an interesting cases study. Over the past 15 years, countries have adopted a wide range of policies that can be divided into four categories—permissive, permissive compromise, restrictive compromise, and prohibitive ^15^. In restrictive policy countries, human embryo research, derivation of hESCs from supernumerary embryos, and somatic cell nuclear transfer (SCNT) are usually prohibited. When permitted, research is possible with imported hESC lines or cell lines that were created before the enactment of any embryo research ban. Predictably, the two countries with the most restrictive policies (Italy and Germany) report very low Attraction Scores ( Figure S17) (sheet 20). Attraction Scores are progressively higher as countries’ policies become more permissive. The highest Attraction Scores belong to South Korea and Australia. This conclusion is reinforced by the analysis of a second score (hESC/ESC score), which assesses the relative focus on human embryonic stem cells. This score is the ratio between the number of publications on “human embryonic stem cells” and the number of publications on “embryonic stem cells” (searches for these key words were, as usual, in the “Title/Abstract” field). The highest hESC/ESC scores belong to Spain and Australia, in which SNCT and derivation of hESC from supernumerary embryos are permissible, with the score of the highest ranking country (Australia) being 4.7 times higher than the score of the lowest ranking country (Italy). Germany reported the third lowest hESC/ESC score.

The ranking represents three cases of apparent outliers: Canada, Japan, and the United States. Canada and the United States report Attraction Scores that are apparently higher than their policies would intuitively suggest. However, a closer look at the policies of these countries explains their position in the ranking. Canada explicitly permitted research with human pluripotent stem cell research since 2002 and since 2006 legalized research on supernumerary embryos. Federal funding for hESC research has been available since the early 2000s and has been comparatively generous since then as evidenced by the fact that, in 2009, the MaRS Regenerative Medicine 2009 Industry Briefing report ranked Canada 4th (after the United States, the United Kingdom, and South Korea) for government funding for stem cell research ( www.marsdd.com/mars-library/regenerative-medicine-industry-briefing/) ^16^. In 2013, “at least 68 centers” with more than 350 researchers were “investigating stem cells and regenerative medicine” ( http://www.ic.gc.ca/eic/site/lsg-pdsv.nsf/eng/hn01746.html Table 1, 4 (last updated June 18, 2013)) ^17^. The United States, another apparent outlier, is classified as a case of “permissive compromise”. Yet, the reality has been different. Policies were restrictive only at the federal level with the *Dickey*- *Wicker* Amendment and President Bush’s a ban on federal funding for research on certain stem cells. However, many US states funded and promoted a significant amount of stem cell research ^18^, which resulted in a high Attraction Score for both 2008–2012 ( Figure S17) and 2003–2007 (sheet 19). On the other hand of the spectrum, Japan adopted more liberal policies but reports a comparatively low Attraction Score. This is due to the way in which the regulatory requirement for prior approval before using hESC translated into an “excessively burdensome approvals process”, which is often blamed for Japan lagging behind other countries ^19^. In 2010, Kawakami *et al.* ^19^ worried that “[a]lthough direct, quantitative effects are difficult to demonstrate, it seems reasonable that these regulatory delays have presented serious challenges to Japanese researchers working, or seeking to work, in these fields, and ultimately impeded progress and competitiveness”. In addition, Japan has focused its research efforts in regenerative medicine on iPSs rather than hESCs as discussed above. Based on these results, we submit that Attraction Scores reveal a relationship between hESC policies and research output in this area.

### Strengths and limitations of the method

As the other metric methodologies ^1– 6, 9– 11, 20^, the one described in this report presents “pros” and “cons” as well as strengths and weaknesses. Indeed, there are some caveats to take in consideration when using this methodology or interpreting the data.

First, as also shown in Figure S1, the country name is not always present in the affiliation. This would not be a problem if all the countries had the same proportions of publications without the country name in the affiliation. However, it is possible that different countries have different proportions of affiliations without the country name. In any case, we think that this is likely not a significant problem given that the vast majority of the publications have the country name in the affiliation and that it is unlikely that research groups around the world have very different habits when indicating the affiliation details on papers. In any case, small differences (if any) should be fully compensated when calculating the ratios between counts of the same country, as in the case of measuring ratios relative to changes in publication output over time or Attraction Scores.

Second, this method may not be perfectly accurate with regard to countries that are recorded under multiple names including acronyms. If a country name is missing then the count for that country would be underestimated. However, in our study we tried to include all possible country names. For example, the United States was searched including not only searches for “united states” but also for “us” OR “usa” (notably when we searched only for “united states” OR “usa” the volumes of publications were decreased by about 10%). Moreover, in some cases the name/acronym of a country could be the same as other affiliation specifications. This particular case may lead to overestimating the number of publications for that country. For example, the acronym US could be present in affiliations not related to the United States, and therefore, even if we believe that (based also on pilot tests we have performed; data not shown) this effect is conceivably negligible, there might be the possibility that the searches for the United States are slightly overestimated. Also, there are cases in which a name can be both a country and a region of another country. This is the case of Georgia, whose number of publications are likely overestimated because “georgia” may be listed as a sovereign country or a state that is part of the United States.

Third, this method relies on first authors’ affiliation information to attribute publications to a certain country. Given that the first author ordinarily either matches the corresponding author or has the same affiliation country of the corresponding author and that the first author (even when she/he is not the corresponding author) frequently plays a prominent role in the project design or execution, this approach has the advantage of classifying papers based on the effective location of the main ideation and execution of research projects. Furthermore, even if some researchers have multiple affiliations, it is plausible that in many cases the multiple affiliations of a single researcher are from the same country; in addition, the single affiliation listed in PubMed, being the one usually more involved in the execution of the research project that led to that specific publication, is most likely the one that better serves our analytical purposes.

**Figure S18.**
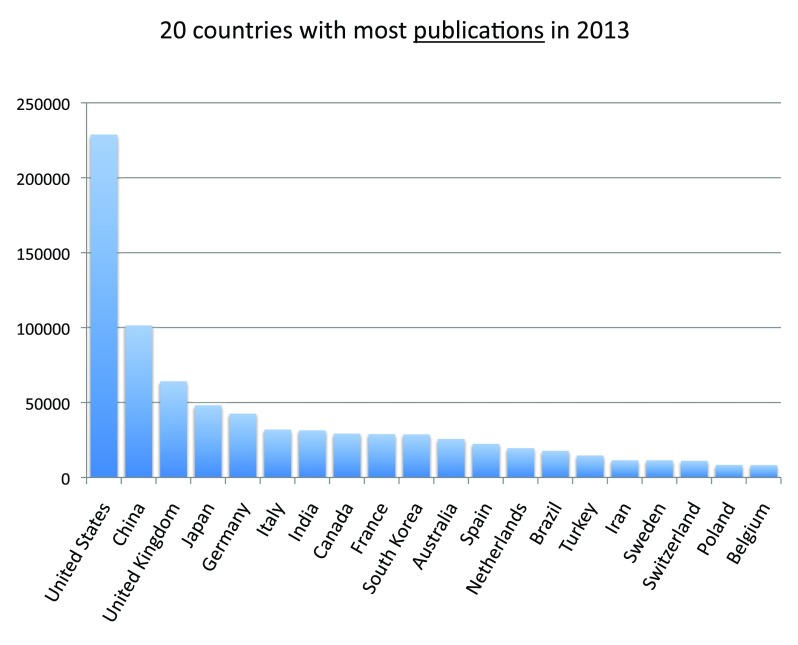
Numbers of publications (reviews excluded) of the 20 countries with the most publications (reviews excluded) in the year 2013. Criteria A.

**Figure S25.**
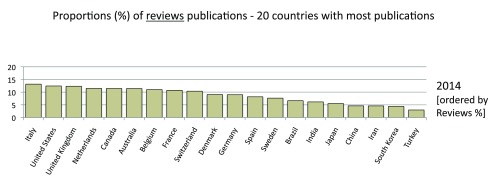
Estimation of the percentages of review publications of the 20 countries with the most publications (reviews excluded) in 2014 [ranked by percentage of reviews]. The percentages were calculated by determining the proportions between the numbers of publications obtained with Criteria A and the numbers obtained with Criteria A but without the exclusion of the reviews.

Thus, this method is useful to determine publication outputs based on leading contributions; however, it is less accurate when all types of contributions need to be taken in account. In this regard, it should be noted that at the beginning of 2014, PubMed started inserting affiliation information for every author of published articles, however without the possibility of limiting the search to first authors ( http://www.ncbi.nlm.nih.gov/books/NBK3827/#pubmedhelp.Affiliation_AD). Therefore, the method used in this paper cannot be used for papers published in 2014 or after. While PubMed’s decision to include information for all authors of a paper is welcomed, it would be desirable to be able again to select affiliation information only for the first (and possibly also specifically for the last) author. In any case, this recent PubMed change offers the possibility to assess the countries’ biomedical publications by taking in consideration all contributing authors, including authors with minor contributions (that are usually placed in the middle of the list of authors). Even if assessing the countries’ biomedical publications based on the first author provides slightly different information from an assessment based on all authors, we expect that the patterns presented in this paper will not substantially change even if the new settings of PubMed were to be used. This is also suggested by the very similar patterns of publications in the years 2008–2012, 2013 (the last year with the old settings) and 2014 (the first year with the new settings) ( Figure S18– Figure S25). At any rate, repeating this study by taking in consideration all authors could be informative to determine which countries have a propensity for leading (i.e. most of ideation and execution) versus assisting (i.e. least of ideation and execution) roles in biomedical research. However, this can only be done in the future, once that enough literature will be archived under the new settings (as of now, still not every currently published paper can be retrieved with the affiliation of all authors).

Fourth, PubMed’s records may be incomplete and thus not perfectly accurate. This is the case of information such as the language in which the paper is written and whether the paper is a review.

Fifth, even if we excluded reviews from our counts (except where otherwise indicated), we did not exclude all publications that are not based on original research. This is the case of comments and editorials. Our choice was motivated by reasons of simplicity and because we believed that PubMed’s tagging of publications as “letters” or “editorials” could be not fully accurate and possibly unequal among countries. However, we believe this choice does not have an impact on data because it is plausible that these types of publications do not significantly affect the relative differences between countries as suggested also by the fact that, when we chose to count only the publications published as “article journal” (criteria C), we did not observe substantial differences with the standard criteria (criteria A) (sheet 3).

Sixth, the quality (however one defines “quality” in this context) of publications is not taken in account. This methodology quantifies the research output by determining the numbers of publications or ratios between numbers of publications and other variables. Proxies for the quality of the papers, such as numbers of citations, numbers of downloads, and impact factors of the journals, are not taken in consideration. Even if indexes based on the quality of research have been already proposed ^1– 6, 10, 11, 20^, we argue that any means of measuring the quality of science will always be partial and controversial and for this reason it will always be useful to take in consideration also (or, in specific circumstances, only) the total volumes of publications. Regardless, the methods and results presented in this paper are not to be intended as neither exclusive nor the perfect means of assessing countries’ research output. In fact, they are better seen as complementary to other methods and results.

Seventh, though PubMed is a search engine that is based on authoritative and comprehensive databases, it might not retrieve some publications. For this reason, it will be important to confirm these results by analyzing also other authoritative and comprehensive databases like Scopus (Elsevier) and Web of Science (Thomson Reuters) that might contain a few publications not retrieved by PubMed. In any case, given the comprehensiveness and breadth of PubMed databases, we believe that the results presented in this paper are a *bona fide* representation of the whole literature. Moreover, the fact that the paper is based on a database that is free and easily accessible by everyone in the world, certainly adds value to the results as it provides tools that can be freely used by anyone and it facilitates readers access to the underlying data, reproducibility, and comparison with our approaches.

If the limitations are taken into account, we believe that the methodology and information presented in this paper can be used, in conjunction with other metrics, to assess research systems in terms of publication output. In particular, we think that the volumes of publications, the relative changes in time, and the Attraction Scores described here provide valuable and unique information about the biomedical and biological research systems of countries. This will assist scholars in studying research systems and policymakers in designing policies to improve scientific production and its benefits to society.

## Data availability

The data referenced by this article are under copyright with the following copyright statement: Copyright: © 2015 Xu Q et al.

Data associated with the article are available under the terms of the Creative Commons Zero "No rights reserved" data waiver (CC0 1.0 Public domain dedication). 

figshare: Biomedical publication and Attraction Score data based on PubMed searches, http://dx.doi.org/10.6084/m9.figshare.1246898 ^21^

## Figures and Tables

**Figure S19.**
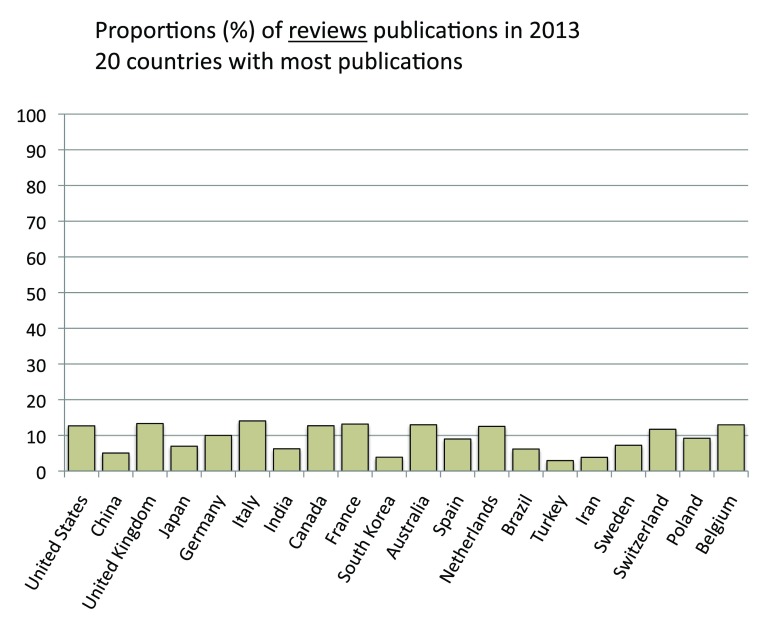
Estimation of the percentages of review publications of the 20 countries with the most publications (reviews excluded) in the year 2013. The percentages were calculated by determining the proportions between the numbers of publications obtained with Criteria A and the numbers of publications obtained with Criteria A but without the exclusion of the reviews.

**Figure S20.**
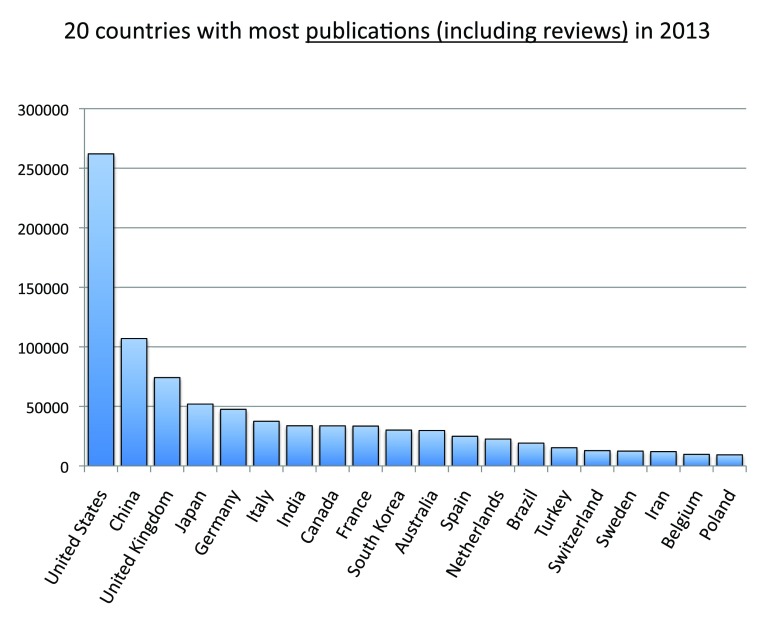
Numbers of publications (reviews included) of the 20 countries with the most publications in the year 2013. Criteria A (without reviews exclusion).

**Figure S21.**
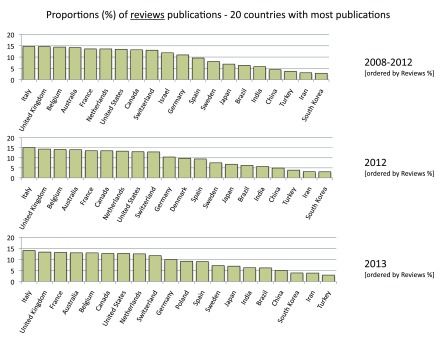
Estimation of the percentages of review publications of the 20 countries with the most publications (reviews excluded) in the indicated years [ranked by percentage of reviews]. The percentages were calculated by determining the proportions between the numbers of publications obtained with Criteria A and the numbers of publications obtained with Criteria A but without the exclusion of the reviews.

**Figure S22.**
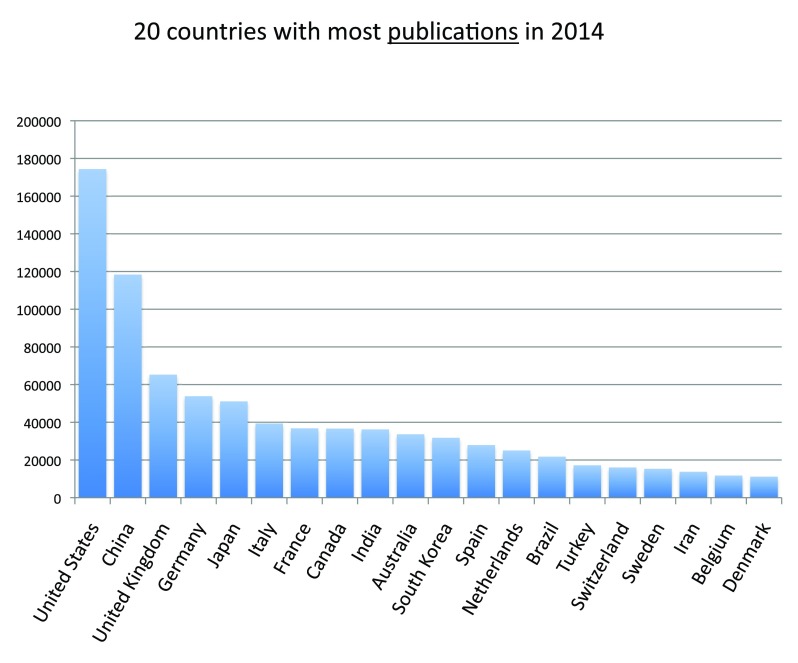
Numbers of publications (reviews excluded) of the 20 countries with the most publications (reviews excluded) in the year 2014. Criteria A. Note that starting from 2014 PubMed reports affiliation information not only of the first author (see Methods and Discussion sections for a correct interpretation of 2014 counts).

**Figure S23.**
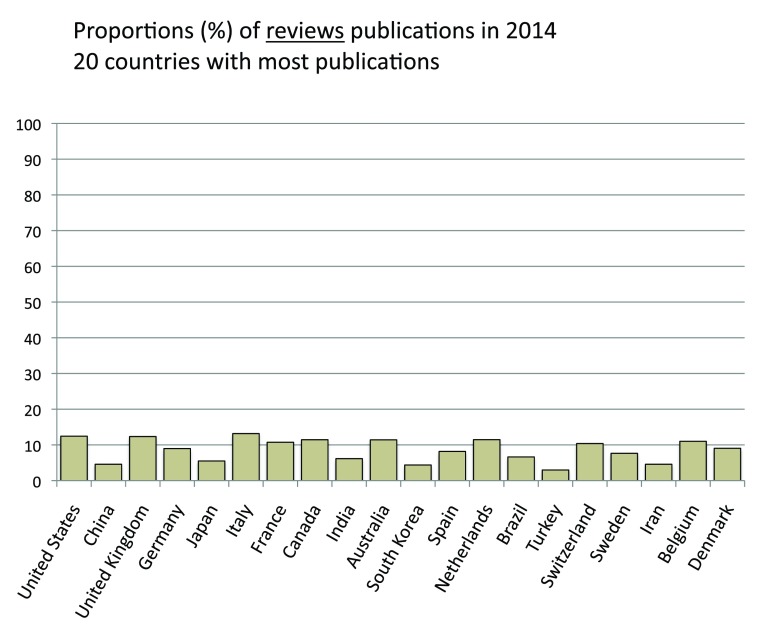
Estimation of the percentages of review publications of the 20 countries with the most publications (reviews excluded) in the year 2014. The percentages were calculated by determining the proportions between the numbers of publications obtained with Criteria A and the numbers of publications obtained with Criteria A but without the exclusion of reviews.

**Figure S24.**
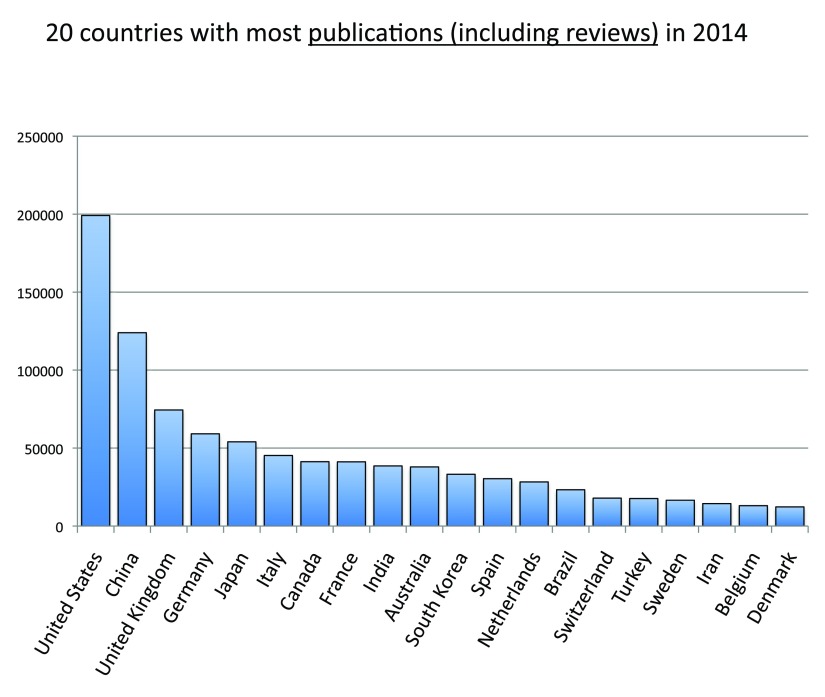
Numbers of publications (reviews included) of the 20 countries with the most publications in the year 2014. Criteria A (without reviews exclusion).

## References

[1] López-Illescas C de Moya Anegón F Moed HF: Comparing bibliometric country-by-country rankings derived from the Web of Science and Scopus: the effect of poorly cited journals in oncology. *J Inf Sci.* 2009;35:244–256. 10.1177/0165551508098603

[2] Nejati A Jenab SMH: A two-dimensional approach to evaluate the scientific production of countries (case study: the basic sciences). *Scientometrics.* 2010;84(2):357–364. 10.1007/s11192-009-0103-1

[3] Anonymous. SCImago Journal & Country Rank. Reference Source

[4] Anonymous. The Research & Innovation Performance of the G20. Reference Source

[5] Anonymous. Knowledge, networks and nations Global scientific collaboration in the 21st century. Reference Source

[6] Jazayeri SB Alavi A Rahimi-Movaghar V: Situation of medical sciences in 50 top countries from 1996 to 2010--based on quality and quantity of publications. *Acta Med Iran.* 2012;50(4):273–278. 22592578

[7] Rahman M Fukui T: Biomedical research productivity: factors across the countries. *Int J Technol Assess Health Care.* 2003;19(1):249–252. 10.1017/S0266462303000229 12701955

[8] Rahman M Fukui T: Biomedical publication--global profile and trend. *Public Health.* 2003;117(4):274–280. 10.1016/S0033-3506(03)00068-4 12966750

[9] Loria A Arroyo P: Language and country preponderance trends in MEDLINE and its causes. *J Med Libr Assoc.* 2005;93(3):381–385. 16059428 PMC1175804

[10] Falagas ME Pitsouni EI Malietzis GA: Comparison of PubMed, Scopus, Web of Science, and Google Scholar: strengths and weaknesses. *FASEB J.* 2008;22(2):338–342. 10.1096/fj.07-9492LSF 17884971

[11] Hightower C Caldwell C: Shifting Sands: Science Researchers on Google Scholar, Web of Science, and PubMed, with Implications for Library Collections Budgets. *Issu Sci Technolog Librarianship.* 2010 10.5062/F4V40S4J

[12] Takahashi K Yamanaka S: Induction of pluripotent stem cells from mouse embryonic and adult fibroblast cultures by defined factors. *Cell.* 2006;126(4):663–676. 10.1016/j.cell.2006.07.024 16904174

[13] Cyranoski D: Japan ramps up patent effort to keep iPS lead. *Nature.* 2008;453(7198):962–963. 10.1038/453962a 18563108

[14] Song P Inagaki Y Sugawara Y: Perspectives on human clinical trials of therapies using iPS cells in Japan: reaching the forefront of stem-cell therapies. *Biosci Trends.* 2013;7(3):157–158. 10.5582/bst.2013.v7.3.157 23836040

[15] Moon S Cho SB: Differential impact of science policy on subfields of human embryonic stem cell research. *PLoS One.* 2014;9(4):e86395. 10.1371/journal.pone.0086395 24717403 PMC3981698

[16] Anonymous. Ontario’s MaRS Regenerative Medicine 2009 Industry Briefing report.

[17] Anonymous Government of Canada, Canadian Asset Map for Stem Cell and Regenerative Medicine: Overview of Stem Cell and Regenerative Medicine Research in Canada. Reference Source

[18] Johnson JA Williams ED: Stem cell research: State initiatives.2006 Reference Source

[19] Kawakami M Sipp D Kato K: Regulatory impacts on stem cell research in Japan. *Cell Stem Cell.* 2010;6(5):415–418. 10.1016/j.stem.2010.04.010 20452315

[20] Anonymous. Introducing the index. *Nature.* 2014;515:S52–S53. 10.1038/515S52a 25390142

[21] Xu Q Boggio A Ballabeni A: Biomedical publication and Attraction Score data based on PubMed searches. *Figshare.* 2014 Data Source

