# Supplementary material for: Countries’ Biomedical Publications and Attraction Scores. A PubMed-based assessment
Source: F1000Res. 2015 Aug 11;3:292. Originally published 2014 Dec 1. [Version 2] doi: 10.12688/f1000research.5775.2 (PMC4566288; doi:10.12688/f1000research.5775.2)
Supplement: Peer review history

# Peer review: Referee response for version 2

**DOI:** 10.5256/f1000research.7420.r9939

**Published:** 2015-08-18

**Authors:** Youngim Jung

**Affiliations:** 1 Korea Institute of Science and Technology Information, Daejeon, Korea, South

My suggestions have been considered and all my questions positively answered.

I agree that this article should be indexed.

I have read this submission. I believe that I have an appropriate level of expertise to confirm that it is of an acceptable scientific standard.

# Peer review: Referee response for version 1

**DOI:** 10.5256/f1000research.6174.r9097

**Published:** 2015-07-02

**Authors:** Youngim Jung

**Affiliations:** 1 Korea Institute of Science and Technology Information, Daejeon, Korea, South

This paper analyzed the volume of publications in the field of life sciences by country and continent in order to better understand regional differences in life sciences research infrastructure. The authors additionally allocated scores to quantify focal areas of interest and examined the potential use of these scores by exploring the relationship between Attraction Score and research policy on hESCs. 

This report is interesting, providing a detailed analysis with a graphical representation of data that facilitates a smooth understanding of the study's conclusions. 

However, as the first reviewer has mentioned, a limitation seems to be present in terms of the data source used for the study. PubMed is a comprehensive and authoritative database but is generally unable to provide an exhaustive data package for scientometric studies. Although the authors have claimed that the study covers the most recent, diverse and refined research output (via PubMed), the number of publications is likely to be only illustrative of the quantitative aspect of the research output. Similarly, the affiliation of the first author provides partial information on the contribution of each country to the publication. Instead, WoS or SCOPUS may be more useful data sources, allowing analysis of the citations and journal impact factors for all authors, as shown in previous studies. 

In addition, whether the Attraction Scores designated by the authors distinguish between the proportion of studies focused on the topic of interest and the entire number of publications remains open to speculation. 

Finally, the 'correlation analysis' referred to in this study is not an actual correlation analysis (which is a statistical term). It is recommendable to change the term in order to avoid confusion for the reader.

I have read this submission. I believe that I have an appropriate level of expertise to confirm that it is of an acceptable scientific standard, however I have significant reservations, as outlined above.

# Response

**Published:** 2015-08-01

**Authors:** Andrea Ballabeni

**Affiliations:** Harvard School of Public Health, USA

We would like to thank Professor Youngim Jung for taking the time to read the paper and provide their helpful feedback.

Here we provide our responses to their comments:

We agree with the referee (as well as with the other referee) that, although PubMed is a search engine based on comprehensive and authoritative databases, some published papers might not be retrieved by searching in these databases. Having said this, we think that given the comprehensiveness and breadth of the PubMed system, the results presented in this paper are a *bona fide* representation of the literature. In any case, we agree that it will be important to crosscheck these data with data obtained by using other databases such as Scopus (Elsevier) and Web of Science (Thomson Reuter). To address this point, we have added the following paragraph to the Discussion section:

*"Seventh, though PubMed is a search engine that is based on authoritative and comprehensive databases, it might not retrieve some publications. For this reason, it will be important to confirm these results by analyzing also other authoritative and comprehensive databases like Scopus (Elsevier) and Web of Science (Thomson Reuters) that might contain a few publications not retrieved by PubMed. In any case, given the comprehensiveness and breadth of PubMed databases, we believe that the results presented in this paper are a bona fide representation of the whole literature. Moreover, the fact that the paper is based on a database that is free and easily accessible by everyone in the world, certainly adds value to the results as it provides tools that can be freely used by anyone and it facilitates readers access to the underlying data, reproducibility, and comparison with our approaches."*

           

Moreover, in order to stress the point that our method and results are based on PubMed, we have slightly changed the title of the paper.

We agree with the referee that the analysis based on the first author has, together with pros, some cons; for this reason we think that it will be useful to complement this analysis with analyses that include all authors. We have therefore added the following paragraph to the Discussion section:

*"In any case, this recent PubMed change offers the possibility to assess the countries’ biomedical publications by taking in consideration all contributing authors, including authors with minor contributions (that are usually placed in the middle of the list of authors). Even if assessing the countries’ biomedical publications based on the first author provides slightly different information from an assessment based on all authors, we expect that the patterns presented in this paper will not substantially change even if the new settings of PubMed were to be used. At any rate, repeating this study by taking in consideration all authors could be informative to determine which countries have a propensity for leading (i.e. most of ideation and execution) versus assisting (i.e. least of ideation and execution) roles in biomedical research. However, this can only be done in the future, once that enough literature will be archived under the new settings (as of now, still not every currently published paper can be retrieved with the affiliation of all authors)."*

We agree with the referee that analyses of citations and impact factors as well as of other putative quality indicators are not present in this paper. However, this was beyond the purposes of our research and we have already acknowledged this in the first version of the paper with the following paragraph of the Discussion section:

*"Sixth, the quality (however one defines “quality” in this context) of publications is not taken in account. This methodology quantifies the research output by determining the numbers of publications or ratios between numbers of publications and other variables. Proxies for the quality of the papers, such as numbers of citations, numbers of downloads, and impact factors of the journals, are not taken in consideration. Even if indexes based on the quality of research have been already proposed ^1^^– ^^6^^, ^^10^^, ^^11^^, ^^20^ , we argue that any means of measuring the quality of science will always be partial and controversial and for this reason it will always be useful to take in consideration also (or, in specific circumstances, only) the total volumes of publications."*

Moreover, we have now added the following paragraph:

*"Regardless, the methods and results presented in this paper are not to be intended as neither exclusive nor the perfect means of assessing countries’ research output. In fact, they are better seen as complementary to other methods and results."*

Regarding the comment of the referee on the “Attraction Scores”, we do not claim they are a perfect means of measuring the relative focus towards certain topics or areas of research. To avoid any ambiguity on this point we have made a few edits to the text. In particular, the term “assess” has been replaced with “appraise” or “study”. 

We agree with the referee that the term “correlation analysis’ can create confusion. For this reason, as suggested by the referee, we have changed the term throughout the manuscript. In same cases we have substituted it with the term “connection” while in other cases we have replaced it with the term “relationship”.

We would like to thank again Professor Youngim Jung for taking the time to read our paper and provide her helpful feedback. We hope we have addressed all her reservations.

# Response

**Published:** 2015-08-12

**Authors:** Andrea Ballabeni

**Affiliations:** Harvard School of Public Health, USA

We would also like to bring to the attention of our reviewers (Dr Vafa Rahimi Movaghar and Dr Youngim Jung) that we have updated figure S22 and added new figures S23-S25. In these figures we provide an analysis of year 2014. In particular, we observed that the patterns of publications were similar in the years 2008–2012, 2013 (the last year with the old settings regarding the affiliation) and 2014 (the first year with the new settings regarding the affiliation). We have accordingly slightly updated the paragraph added to the Discussion section:

*“In any case, this recent PubMed change offers the possibility to assess the countries’ biomedical publications by taking in consideration all contributing authors, including authors with minor contributions (that are usually placed in the middle of the list of authors). Even if assessing the countries’ biomedical publications based on the first author provides slightly different information from an assessment based on all authors, we expect that the patterns presented in this paper will not substantially change even if the new settings of PubMed were to be used. This is also suggested by the very similar patterns of publications in the years 2008–2012, 2013 (the last year with the old settings) and 2014 (the first year with the new settings) (Figure S18–Figure S25). At any rate, repeating this study by taking in consideration all authors could be informative to determine which countries have a propensity for leading (i.e. most of ideation and execution) versus assisting (i.e. least of ideation and execution) roles in biomedical research. However, this can only be done in the future, once that enough literature will be archived under the new settings (as of now, still not every currently published paper can be retrieved with the affiliation of all authors).”*

# Peer review: Referee response for version 1

**DOI:** 10.5256/f1000research.6174.r7052

**Published:** 2014-12-16

**Authors:** Vafa Rahimi Movaghar

**Affiliations:** 1 Research Centre for Neural Repair, University of Tehran, Tehran, Iran

I read the paper of Qinyi Xu *et al.* entitled "Countries’ Biomedical Publications and Attraction Scores" with great interest. Authors evaluated biomedical publications of all countries based on the PubMed and performed great job on different criteria to assess changes during different time periods based on the country of affiliation of the first authors especially during the five-year time period of 2008-2012 versus 2003-2007. The study is an addition to the literature and fruitful. I would recommend reading the paper to all medical scientists in the world. However, there are some concerns. PubMed is not the only search engine to be evaluated. There are other valuable sources such as ISI, Embase and Scopus which may change the results of the study. We would know that there are many journals indexed in the above-mentioned search engines especially written in the developing countries. On the other hand, quantity evaluation does not show the real scientific situation of the countries. I myself prefer the citation without self-citation for the best index of quality assessment. Other indices such as H-index reveal the mixture of quantity and quality assessment. The affiliation of the first author is acceptable, but not ideal to show the comprehensive situation of the countries. All authors’ affiliation or at least first and corresponding authors affiliations are better replacement in the future studies. Authors mentioned that they reported original articles. Therefore, they deleted reviews. However, I would recommend not deleting the review articles unless simultaneously deleting letters and correspondences.

I have read this submission. I believe that I have an appropriate level of expertise to confirm that it is of an acceptable scientific standard.

# Response

**Published:** 2014-12-19

**Authors:** Andrea Ballabeni

**Affiliations:** Harvard School of Public Health, USA

We would like to thank Professor Vafa Rahimi Movaghar for reviewing our article. We are glad that the reviewer appreciated our study.

Here are some preliminary answers to his few concerns.

1. The reviewer is right about the fact that PubMed is not the only source of biomedical publications and that some journals are not indexed in PubMed. We will certainly add remarks on this point in the revised version of the manuscript, which we will submit after receiving feedback from all reviewers.
2. The reviewer mentions about the fact that our method measures only the quantity of publications. He is right but we have already addressed this issue in the Discussion section of the paper. In particular, we do not claim that our data can fully measure the efficiency of countries in biomedical research. Our data and methods need to be complemented with other data and methods. Our revised draft will certainly emphasize this point further.
3. The reviewer mentions the limitations of using first authors’ affiliations. His comment has merit and in fact we have already acknowledged the pros and cons of this approach in the Discussion section.
4. The reviewer mentions that it would be better to include reviews publications in the publication count. While we decided not to include reviews in the majority of calculations, we included them in some other calculations. We have observed that reviews amount to a rather small proportion of publications and consequently their inclusion does not substantially affect the rankings (this was done for years 2012 and 2013). We discuss this issue in the Results and Discussion sections.

 

We would like to thank again Professor Vafa Rahimi Movaghar for taking the time to read the article and providing his feedback

Sincerely

The authors

# Response

**Published:** 2015-08-01

**Authors:** Andrea Ballabeni

**Affiliations:** Harvard School of Public Health, USA

We would like to confirm our first response to Professor Vafa Rahimi Movaghar. We have waited for a second review before revising the paper based on the reviewers’ suggestion. We now provide a revised version based on the feedback received from both referees.

We would like to thank again professor Vafa Rahimi Movaghar for taking the time to read and comment our paper and for this helpful feedback.
